# Blood–brain barrier dysfunction in aging is mediated by brain endothelial senescence

**DOI:** 10.1111/acel.14270

**Published:** 2024-08-15

**Authors:** João P. Novo, Lucy Gee, Carolina A. Caetano, Inês Tomé, Andreia Vilaça, Thomas von Zglinicki, Irina S. Moreira, Diana Jurk, Susana Rosa, Lino Ferreira

**Affiliations:** ^1^ CNC‐UC Center for Neuroscience and Cell Biology University of Coimbra Coimbra Portugal; ^2^ University of Coimbra, Institute for Interdisciplinary Research Doctoral Programme in Experimental Biology and Biomedicine (PDBEB) Coimbra Portugal; ^3^ CIBB—Centre for Innovative Biomedicine and Biotechnology University of Coimbra Coimbra Portugal; ^4^ Department of Physiology and Biomedical Engineering Mayo Clinic Rochester Minnesota USA; ^5^ Faculty of Medicine University of Coimbra Coimbra Portugal; ^6^ Biosciences Institute, Faculty of Medical Sciences Newcastle University Newcastle upon Tyne UK; ^7^ Department of Life Sciences, University of Coimbra Coimbra Portugal

**Keywords:** aging, blood–brain barrier, brain endothelial cells, senescence, senolytics

## Abstract

BBB dysfunction during aging is characterized by an increase in its permeability and phenotypic alterations of brain endothelial cells (BECs) including dysregulation of tight junction's expression. Here we have investigated the role of BEC senescence in the dysfunction of the BBB. Our results suggest that the transition from young to aged BBB is mediated, at least in part by BEC senescence.

## Graphical abstract

The current study shows that the accumulation of senescent brain endothelial cells (BECs) may mediate the transition from a young to an aged BBB. Aged BECs have a higher expression of senescent markers and impaired occludin expression which interferes with BBB function. These phenotypes were partially reverted after the systemic elimination of p16^+^ cells by genetic means. The study contributes for a better understanding of BBB dysfunction during aging.

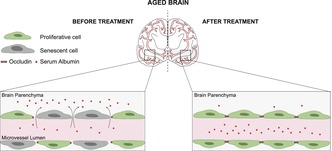

**Abbreviations**
AP: AP20187
BBB: blood–brain barrier
BECs: brain endothelial cells
D+Q: Dasatinib+Quercitin
DEGs: differentially expressed genes
GFAP: Glial fibrillary acidic protein
GLUT‐1: Glucose transporter protein isoform 1
HMGB1: High Mobility Group Box 1
RNAseq: RNA sequencing
SA‐β‐Gal: Senescence associated beta galactosidase

The blood–brain barrier (BBB) is a physical and metabolic barrier formed by brain endothelial cells (BECs) that together with pericytes, astrocytes, neurons, and extracellular matrix form the functional neurovascular unit (Aday et al., 2016; Kaplan et al., 2020). Both human and rodent BBB deteriorate during physiological aging which is characterized at functional level by an increased permeability to serum albumin (Senatorov Jr. et al., 2019) and contrast agents (Montagne et al., 2015). This deterioration is characterized at cellular level by a dysfunction of BECs (dysregulation in the expression of tight‐junctions (Elahy et al., 2015; Ximerakis et al., 2019), alterations in transport systems (Yang et al., 2020) as well as by a decrease in the pericyte coverage of the blood vessels (Bell et al., 2010). Unfortunately, the mechanisms underlying the transition from a young‐and‐healthy to aged‐and‐dysfunctional BBB are not well understood. Our hypothesis is that BEC senescence might be an important contributor of BBB dysfunction. Cell senescence, a hallmark of aging triggered by a variety of stresses, is characterized by an arrested cell cycle and a far‐ranging epigenetic and metabolic rewiring including a distinct secretory phenotype (Gorgoulis et al., 2019), which is also observed in aged postmitotic cells including neurons (von Zglinicki et al., 2021). These cells accumulate during physiological (Jurk et al., 2012; Ogrodnik et al., 2021) and pathological (Bussian et al., 2018) aging in the brain. However, the senescence program in BECs and its impact on BBB structure and function are largely unknown. Here, we investigated the senescence program of BECs using a combination of single‐cell transcriptomic studies and protein analyses in mice brain slices and studied the functional impact of BEC senescence in an in vitro human BBB model and in vivo (Figure 1a).

**FIGURE 1:**
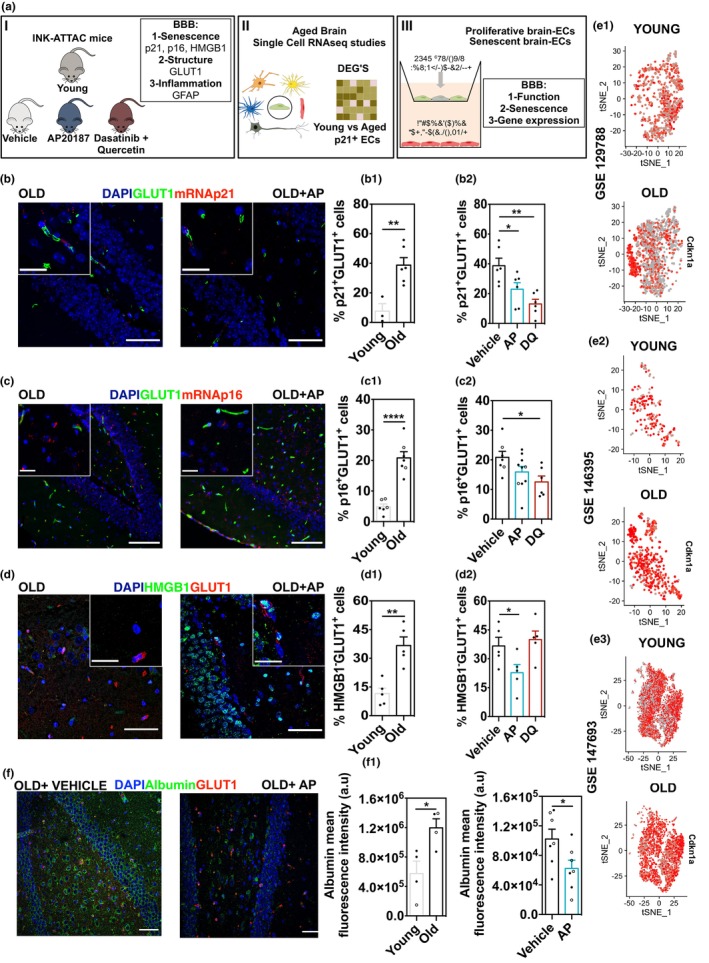
Characterization of the senescence program in BECs. (a) Schematic representation of the study design. (b–d) Representative images for senescence markers p21^+^ (b), p16^+^ (c), and HMGB1^−^ (d) at the BBB in the hippocampus of aged mice with and without treatment. For p16 (red) and p21 (red) RNAish analyses were performed. GLUT1 (green) to stain BECs and HMGB1 were analyzed by immunohistochemistry. Scale bar: (b, d) 50 and 20 μm (crop), (c) 50 and 25 μm (crop). Quantification of the percentage of GLUT1^+^ cells positive for p21 (b.1), p16 (c.1) and negative for HMGB1 (d.1) in young and aged mice. Quantification of the percentage of GLUT1^+^ cells positive for p21 (b.2), p16 (c.2) and negative for HMGB1 (d.2) in aged mice with and without senolytic treatment. Each point represents the mean of all fields (3, 4) analyzed per animal [young: 3 M = male, 3F = female, aged untreated (8 M, 6F), AP (8 M, 2F), DQ (7 M), closed symbol = male and open symbol = female mice]. Results are the mean ± SEM. (f) Representative images of immunostaining for albumin (green) and GLUT1 (red) at the hippocampus of aged INK‐ATTAC mice with and without treatment (AP‐treated and vehicle, respectively). Scale bar: 50 μm. (f.1, f.2) Quantification of the albumin fluorescence intensity in young versus old and vehicle versus AP‐treated animals. Each point represents the mean of all fields (4–6) analyzed per animal [young (3 M, 1F), aged untreated (4 M, 3F), AP (3 M, 4F), closed symbol = male and open symbol = female mice]. Results are the Mean ± SEM. Statistical analysis was performed using an Unpaired Student's *t*‐test (*p < 0.05, *p < 0.05; **p < 0.01). (e) tSNE plots for p21^+^ ECs young versus old from three datasets: GSE129788, GSE146395, and GSE14763. Color gradient code: Grey to red indicate low‐ to high‐expression values.

To characterize the BBB during aging, we used brain sections of young (3 months) and aged C57/BL6 mice (18 months). We selected the hippocampus because it has been shown that BBB permeability increases with aging in humans without cognitive impairment, not observed in other brain regions (e.g., cortical, subcortical, and white matter) (Montagne et al., 2015). In addition, the BBB breakdown in the hippocampus induces human cognitive impairment (Montagne et al., 2015; Nation et al., 2019). Immunohistochemical analysis showed a decrease in the number of GLUT1^+^ (BECs marker) vessels (Figure S1), an increase in the glial fibrillary acidic protein (GFAP) expression (marker of reactive astrocytes) and an increase in the association of GFAP^+^ astrocytes with BECs (Figure S1) in aged versus young brain sections. This phenotype was characterized at functional level by an increase in the BBB permeability to fluorescently labeled serum albumin intravenously injected (Figure S1). Brains of 22 months old mice showed higher fluorescence intensity in comparison with young, suggesting a higher albumin extravasation into the brain.

To investigate the impact of senescence on the BBB permeability we used brain sections from young (3 months) and aged (24–27 months) INK‐ATTC mice, a transgenic mouse model, in which apoptosis of highly p16^Ink4a^‐expressing cells can be induced upon administration of the drug AP20187 (AP), leading to their elimination (Baker et al., 2011; Figure 1a). Previously, we have shown that the impaired cognition in aged mice was improved by treatment with AP or senolytics [Dasatinib + Quercetin (D + Q)] (Ogrodnik et al., 2021). Aged brains showed a significant increase in the number of GLUT1^+^ cells in the hippocampus that stained positive for p21, p16 and were negative for High Mobility Group Box 1 (HMGB1), relatively to young brain (Figure 1b–d). Importantly, frequencies of GLUT1^+^ BECs displaying two out of the three analyzed senescence markers decreased in the hippocampus of animals treated with either AP or DQ, relatively to aged animals without treatment (vehicle). We also found a tendency for: (i) a high number of GLUT1^+^ cells per field in AP‐ and DQ‐treated animals (Figure S3c), (ii) a decrease in GFAP expression and area overlapping with GLUT1^+^ in AP‐treated mice, and (iii) an increase in occludin fluorescence intensity in BECs (VE‐cadherin^+^) (Figure S3d,e). Some of these analyses contained few or no female animals therefore we cannot exclude a sex‐dependent effect.

Next, we investigated whether the systemic elimination of senescent cells could decrease BBB permeability. For this purpose, we used 24–27 months INK‐ATTAC mice treated with AP and analyzed by immunofluorescence the extravasation of serum albumin in brain sections of vehicle and AP mice. Interestingly, sections from AP‐treated animals showed a significantly lower fluorescence intensity for serum albumin (Figure 1e; Figure S2) than control, suggesting that the systemic elimination of senescent cells contributed to a decrease in BBB permeability.

To further confirm the impact of senescence on the BBB in aged mice, we used single‐cell RNA sequencing data from BECs isolated from aged mice (Figure S4) as previously reported (Chen et al., 2020; Ximerakis et al., 2019; Zhao et al., 2020). The RNAseq results indicated an increase in the number of p21^+^ BECs in old versus young brains (Figure 1f). Because p21^+^ cells were also expressed in young BECs, we then investigated the transcriptomic profile in both p21^+^‐ old and p21^+^‐young BECs to identify genes that were differentially expressed in both cell populations. The differentially expressed genes (DEGs) were then intersected with BBB (84 genes; Table S6) and senescence‐enriched genes (279 genes; Table S7; Figure S4d). None of the DEGs were shared by the three analyzed datasets and this might be related to differences in the single‐cell transcriptomic strategies and animal age. However, the results showed a downregulation of some genes related to tight junctions (e.g., *occludin*) and transporters (*Slc* transporters) (Figure S4d). We validated in the hippocampus of aged (24–27 months) brain sections, a decrease in occludin, an important tight junction in BECs (Elahy et al., 2015) (GLUT1^+^ cells) in comparison to cells in young brain sections (Figure 2a).

**FIGURE 2:**
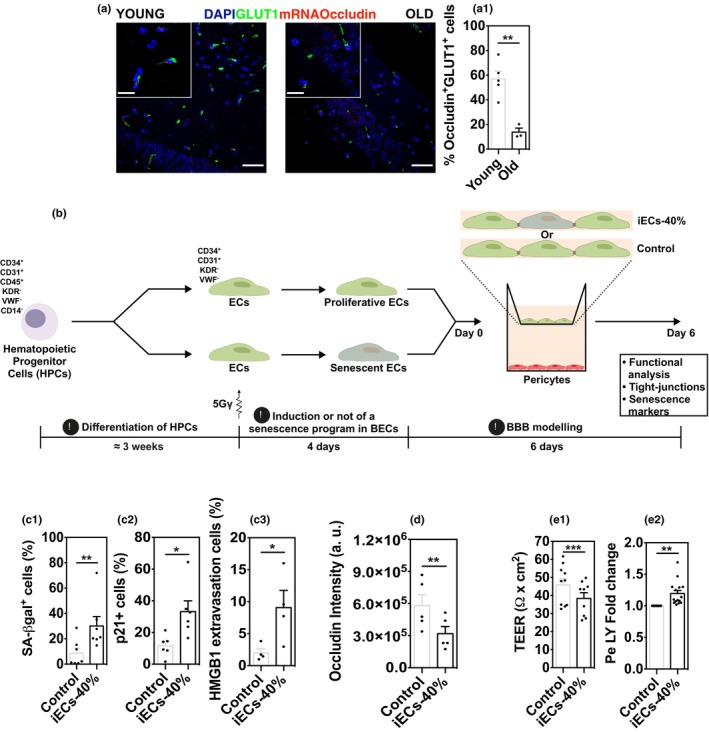
Impact of senescent BECs in the BBB. (a) Representative images of the RNAish analyses for occludin (red) at the BBB in the hippocampus of young and aged mice. BECs were stained for GLUT1 (green) by immunohistochemistry. Scale bar: 50 and 20 μm (crop). (a1) Quantification of the number of GLUT1^+^ cells positive for occludin. Each point represents the mean for all the fields (3, 4) analyzed for each animal (3‐5 M animals, closed symbol). Results are the Mean ± SEM. Statistical analysis was performed using an Unpaired Student's *t*‐test (**p < 0.01). (b) Schematic representation of the experimental design. (c) Senescence program of BECs. Cells were characterized for SA‐β‐galactosidase (c.1), p21 (c.2), and HMGB1 (c.3; cells that present cytoplasmic staining) relative to the total number of nuclei per condition. (d) Occludin mean fluorescence intensity quantification. In C and D, each point represents the mean of the different fields analyzed. Results are the mean ± SEM of 4–7 independent experiments. Statistical analysis was performed using a Paired Student's *t*‐test. (*p < 0.05; **p < 0.01). (e.1) TEER and (e.2) paracellular permeability (Pe) to Lucifer Yellow (LY) measurements. For each experiment, the permeability was normalized to the control (condition without iECs). Results are the Mean ± SEM of 10–15 independent experiments, performed in triplicate. Statistical analysis was performed using a Paired Student's *t*‐test (**p < 0.01; ***p < 0.01).

To better understand the mechanisms by which senescent cells can disturb BBB properties, having in mind the human context, we further developed an in vitro human senescent BBB model based on a previous BBB model developed by us (Cecchelli et al., 2014) to include senescent BECs. The BBB model is established in three steps: In the initial model, CD34^+^ cells were isolated from the mononuclear fraction of human cord blood, differentiated into endothelial cells (ECs) and then co‐cultured for 6 days with pericytes in a transwell system for specification toward BECs. This model has been validated by several laboratories and showed a good correlation with human BBB permeability data (Cecchelli et al., 2014). ECs before specification to BECs, were exposed to gamma radiation (5 Gγ; from now on referred as iECs (irradiated endothelial cells) to initiate a senescence program (Figure 2b and Figures S5–S7)). Approximately 15% of iECs are lost in the first 3 days of culture (Figure S6). In the senescent BBB model, BECs expressed SA‐β‐Gal (30% ± 7.4%), p21 (33% ± 6.8%) and presented HMGB1 extravasion (10% ± 2.7%) (Figure 2c.1,c.2,c.3). Importantly, the functional alterations in the properties of the in vitro BBB model were accompanied by a decrease in the levels of the tight junction protein occludin (Figure 2d), as observed in mouse brain sections. The decrease in the expression of tight junction occludin was more significant in the p21^+^ cells than p21^−^ cells (Figure S7c) suggesting that occludin expression levels is directly associated with BEC senescence. The levels of claudin 5 and VE‐cadherin were not altered by the senescence (data not shown and Figure S7d). This senescent phenotype translated into a BBB model that showed a significant increase in the paracellular permeability to Lucifer Yellow and a decrease in transendothelial electrical resistance (TEER) values as compared to the model with proliferative cells (Figure 2e.1,e.2).

In conclusion, we report here that the transition from young to aged BBB is mediated in part by the induction of senescence in brain endothelial cells. In addition, we show in both mouse brain sections and in vitro human BBB model that aged BECs have impaired occludin expression that may interfere with BBB structure and function.

## EXPERIMENTAL PROCEDURES

Full detailed methods and experimental procedures are available as Appendix S2.

## AUTHOR CONTRIBUTIONS

LF conceived the project. SR and LF designed the experiments. JPN, ACC, IT, ACC, LG, AV, and SR performed data collection. JPN, IT, ACC, LG, AV, TZ, ISM, DJ, SR, and LF analyzed the experiments. JPN, SR, and LF wrote the manuscript. All authors commented and approved the manuscript.

## CONFLICT OF INTEREST STATEMENT

None declared.

## Supporting information


Appendix S1.

Appendix S2.

## Data Availability

The data that support the findings of this study are available from the corresponding author upon reasonable request.
