# Correction to “Blood–brain barrier dysfunction in aging is mediated by brain endothelial senescence”

**DOI:** 10.1111/acel.70067

**Published:** 2025-04-07

Novo, J. P. , Gee, L. , Caetano, C. A. , Tomé, I. , Vilaça, A. , von Zglinicki, T. , Moreira, I. S. , Jurk, D. , Rosa, S. , & Ferreira, L. (2024). Blood–brain barrier dysfunction in aging is mediated by brain endothelial senescence. Aging Cell, 23, e14270. 10.1111/acel.14270 39143890 PMC11488312

In the published version of the above article, we noticed a few typographical errors in Figure 1a(III) and in Figure 2.

The corrected figures are shown below:

**FIGURE 1:**
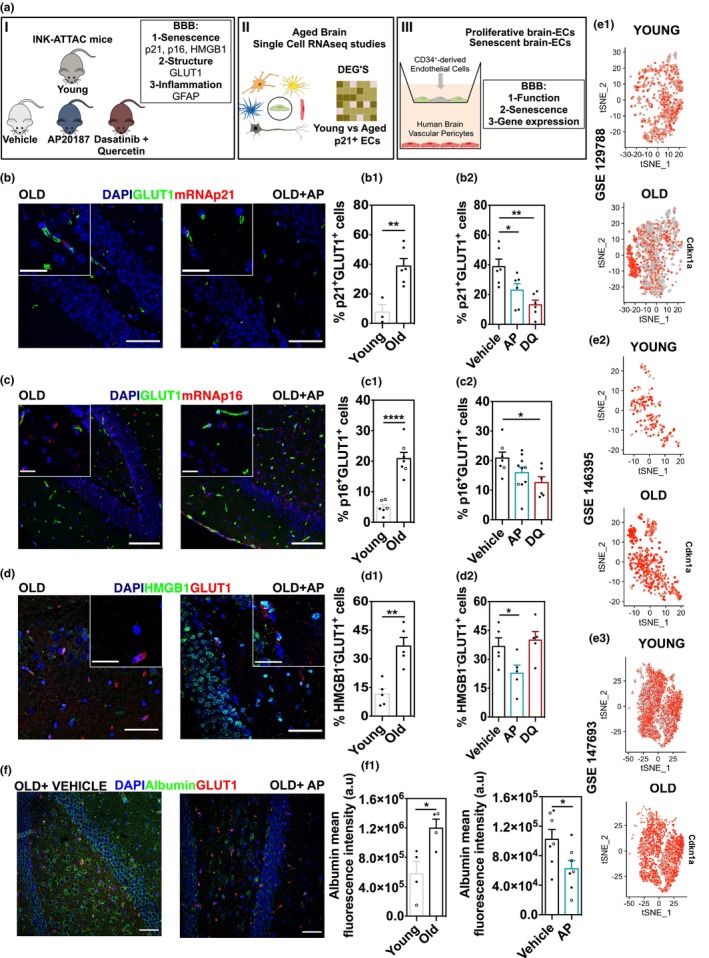


**FIGURE 2:**
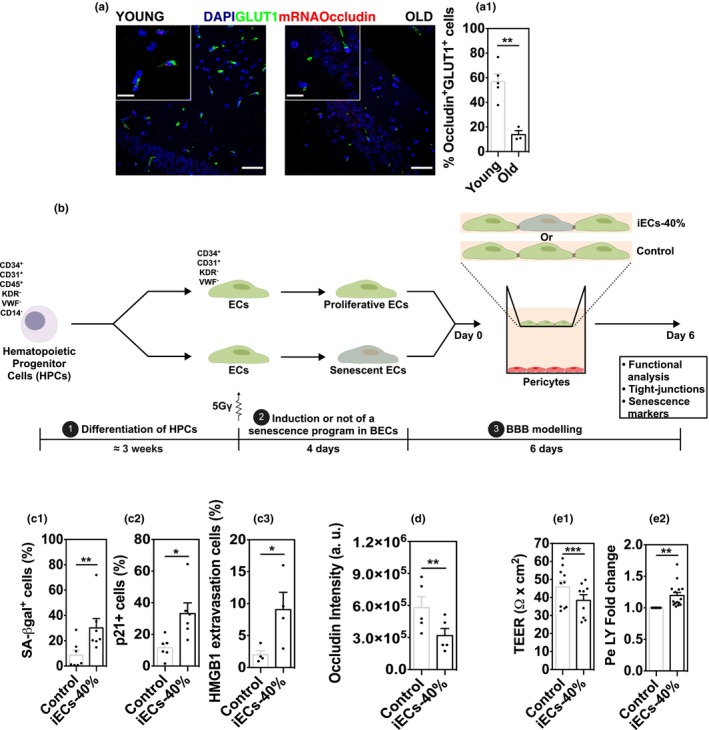


We apologize for this error.

