# Supplementary figures and images for: Blood–brain barrier dysfunction in aging is mediated by brain endothelial senescence
Source: Aging Cell. 2024 Aug 15;23(9):e14270. doi: 10.1111/acel.14270 (PMC11488312; doi:10.1111/acel.14270)

FIG 1

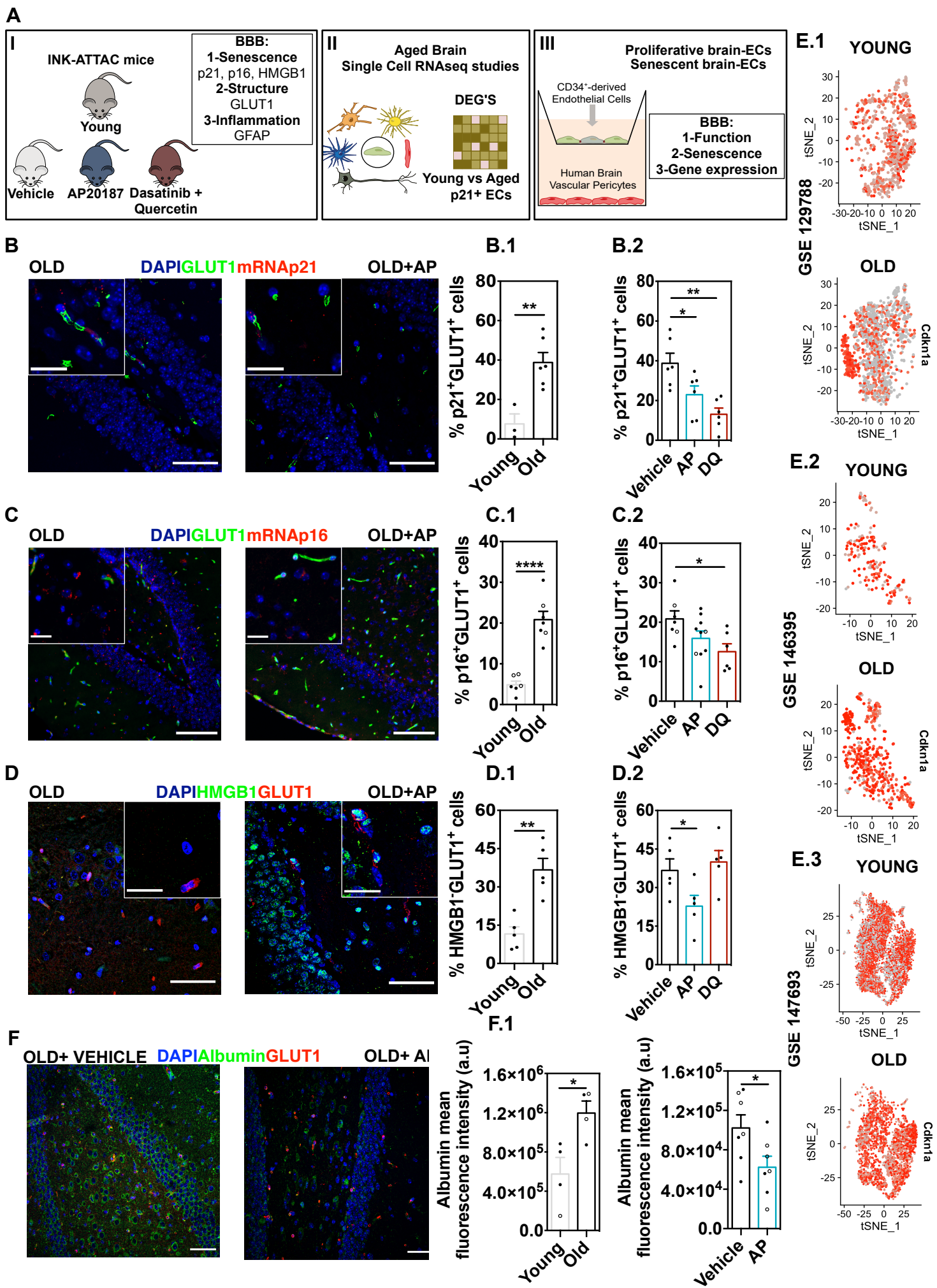

FIG 2

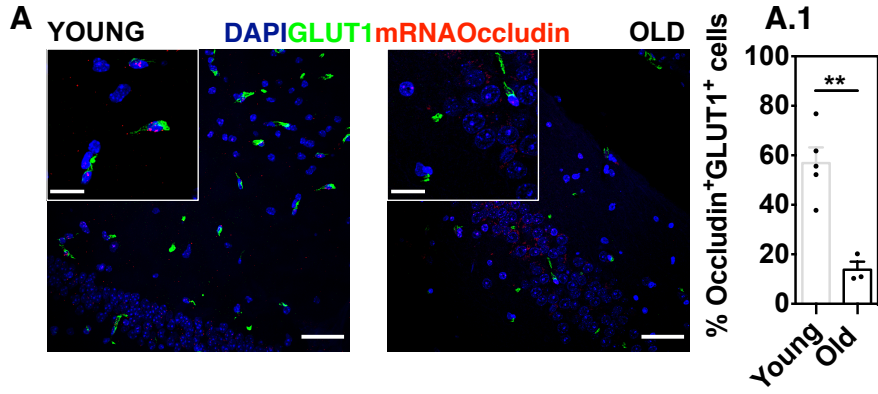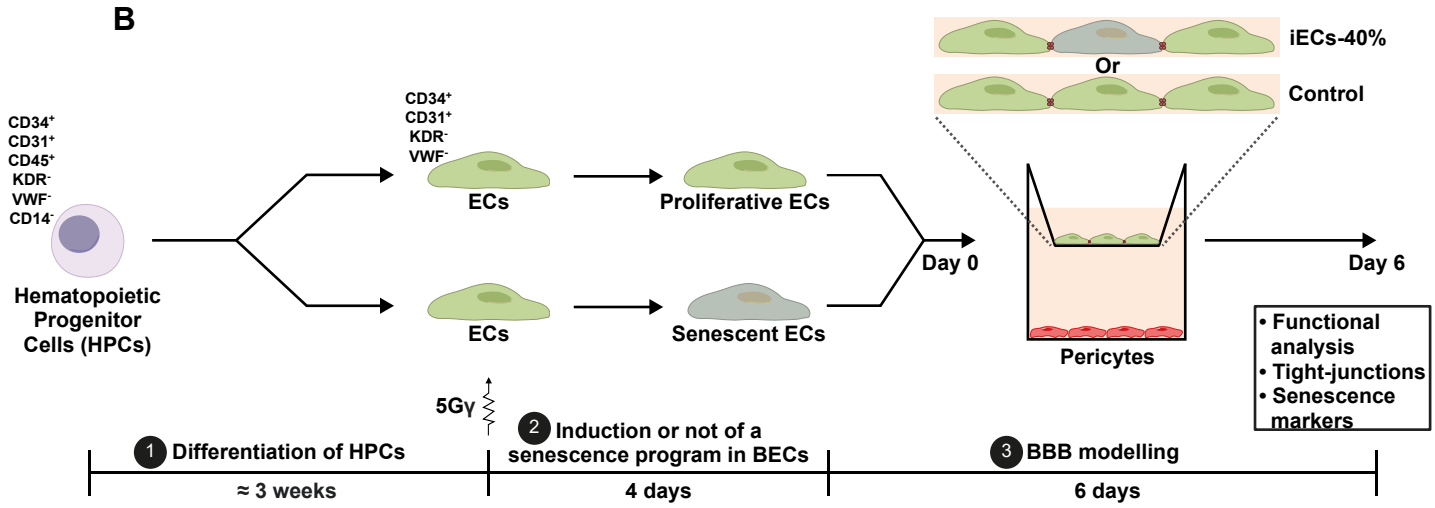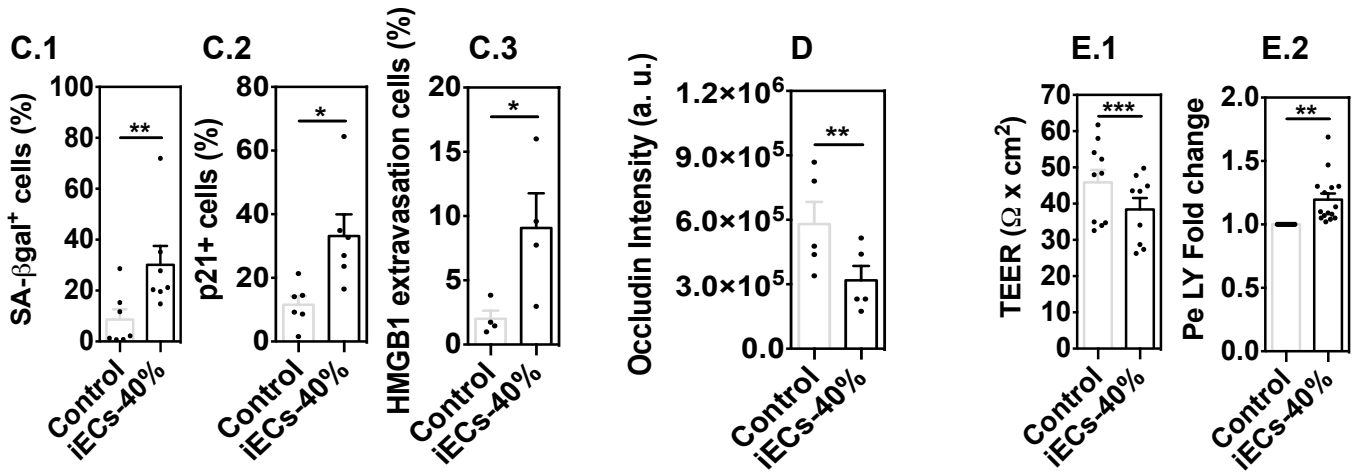

FIG S1

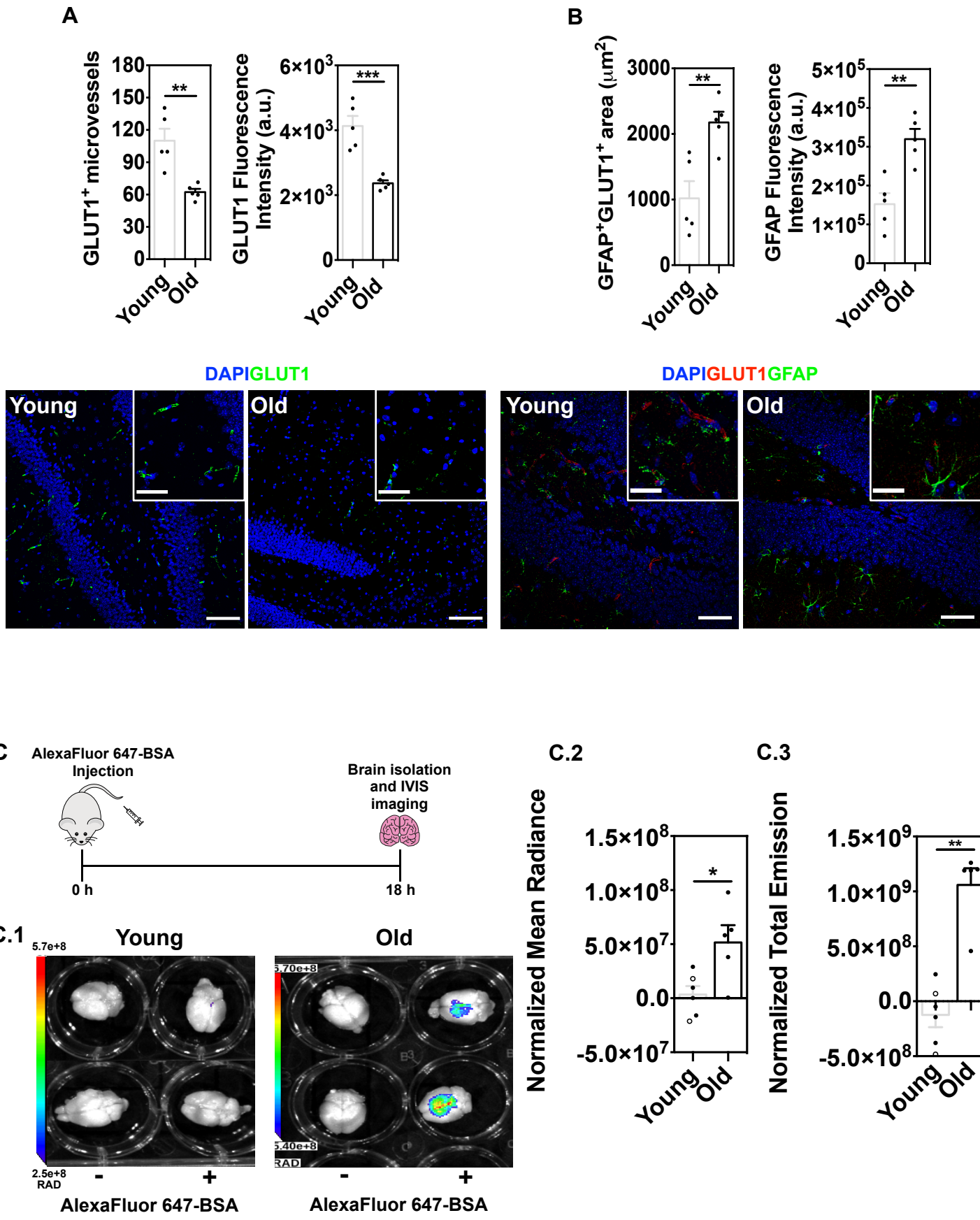

FIG S2

A

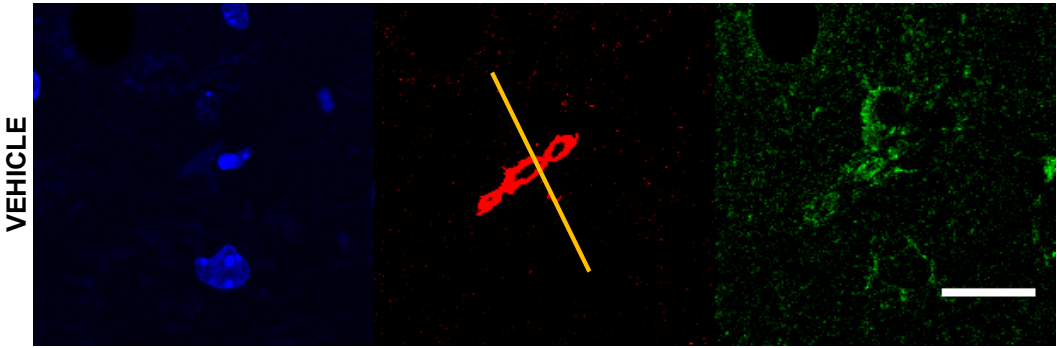

A.1

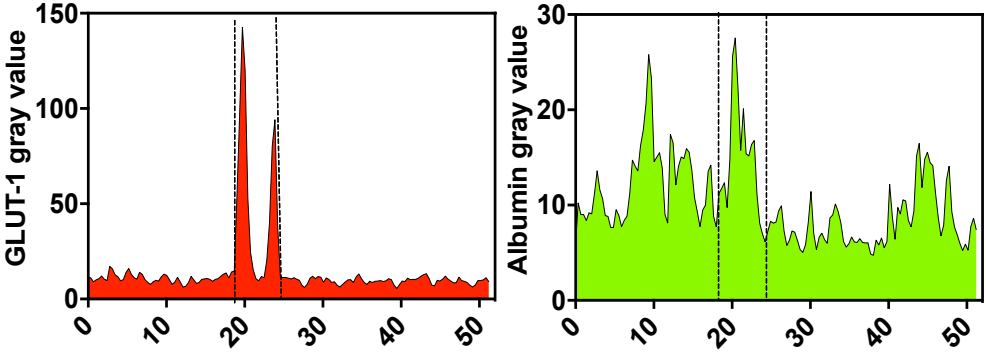

B

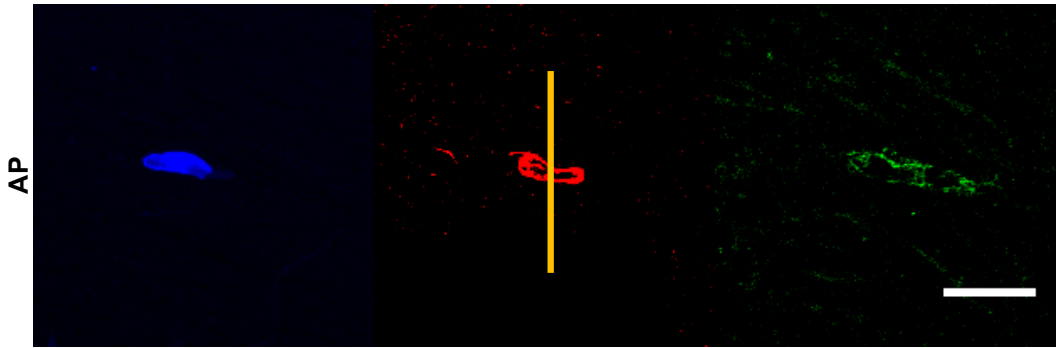

B.1

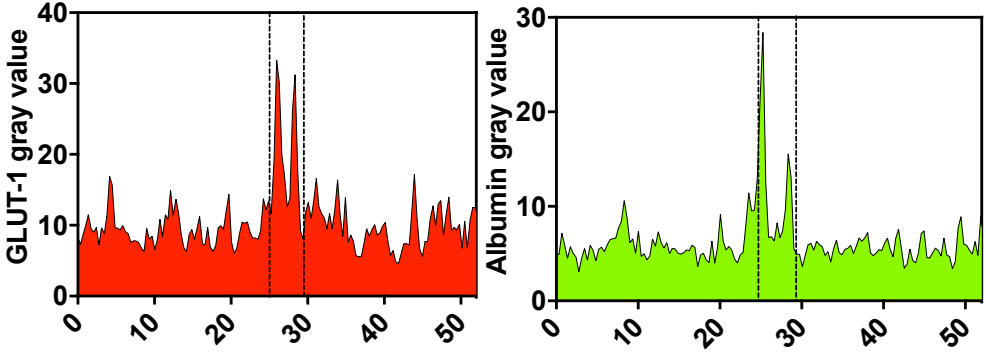

FIG S3

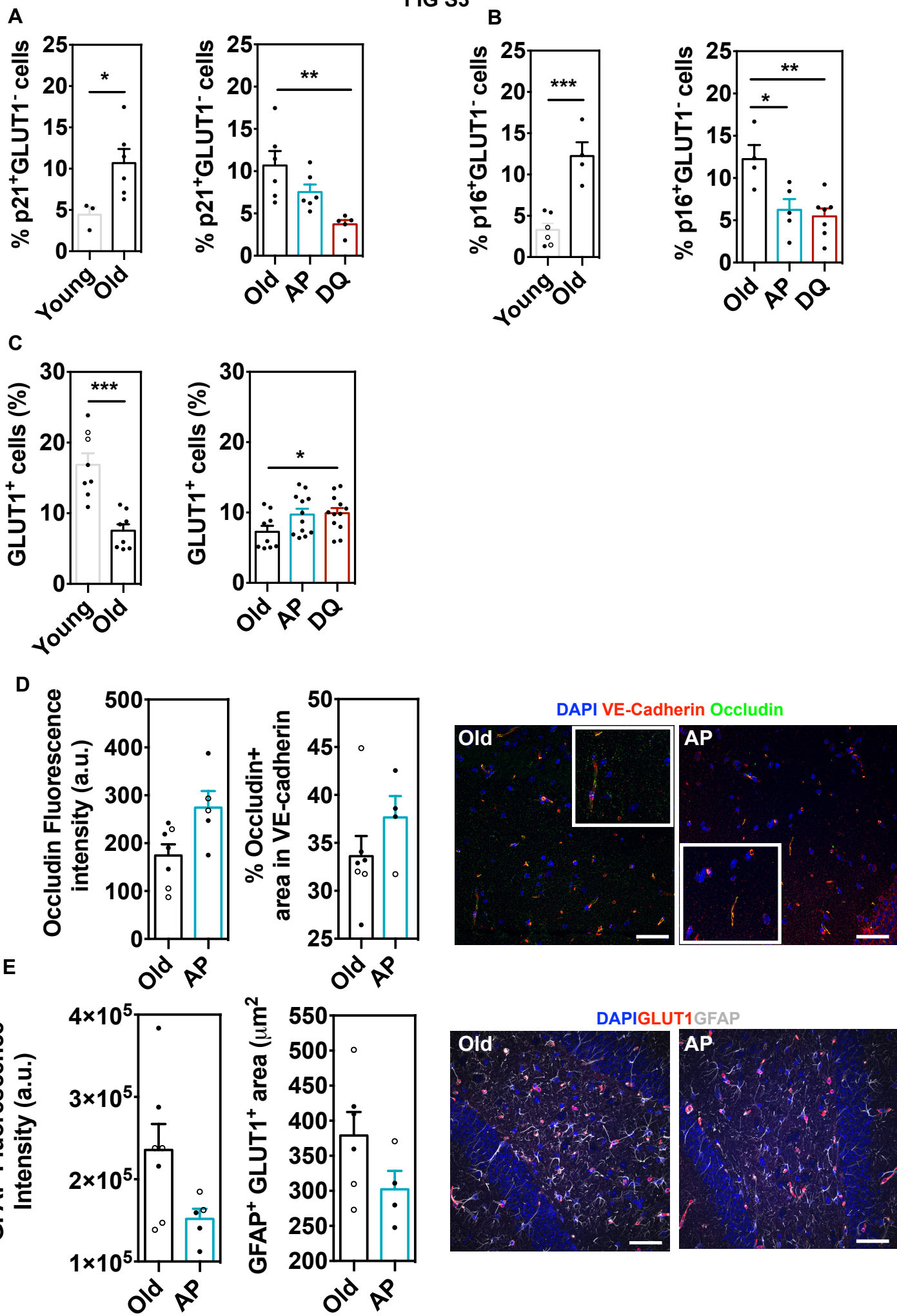

FIG S4

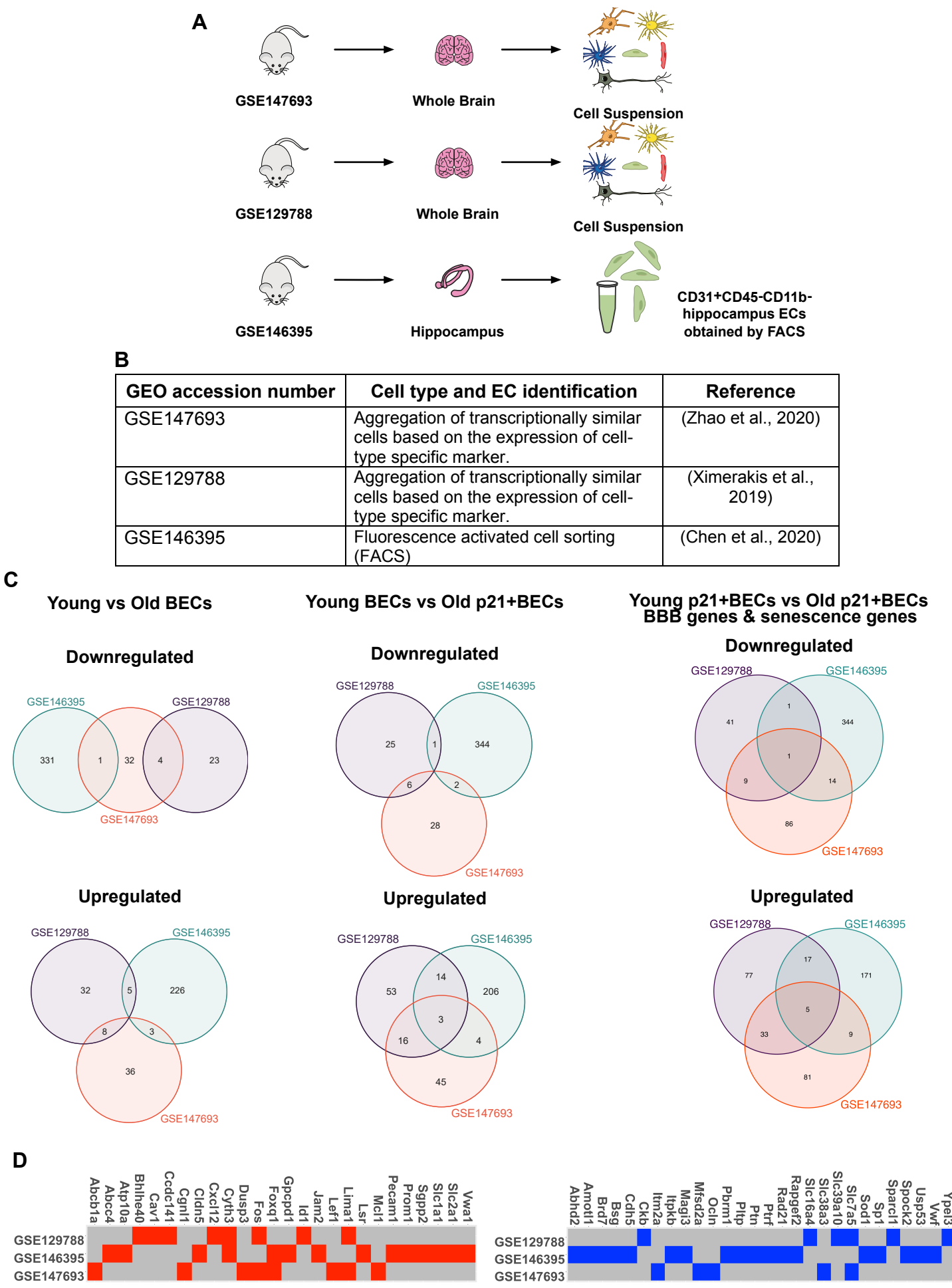

**FIG S5**

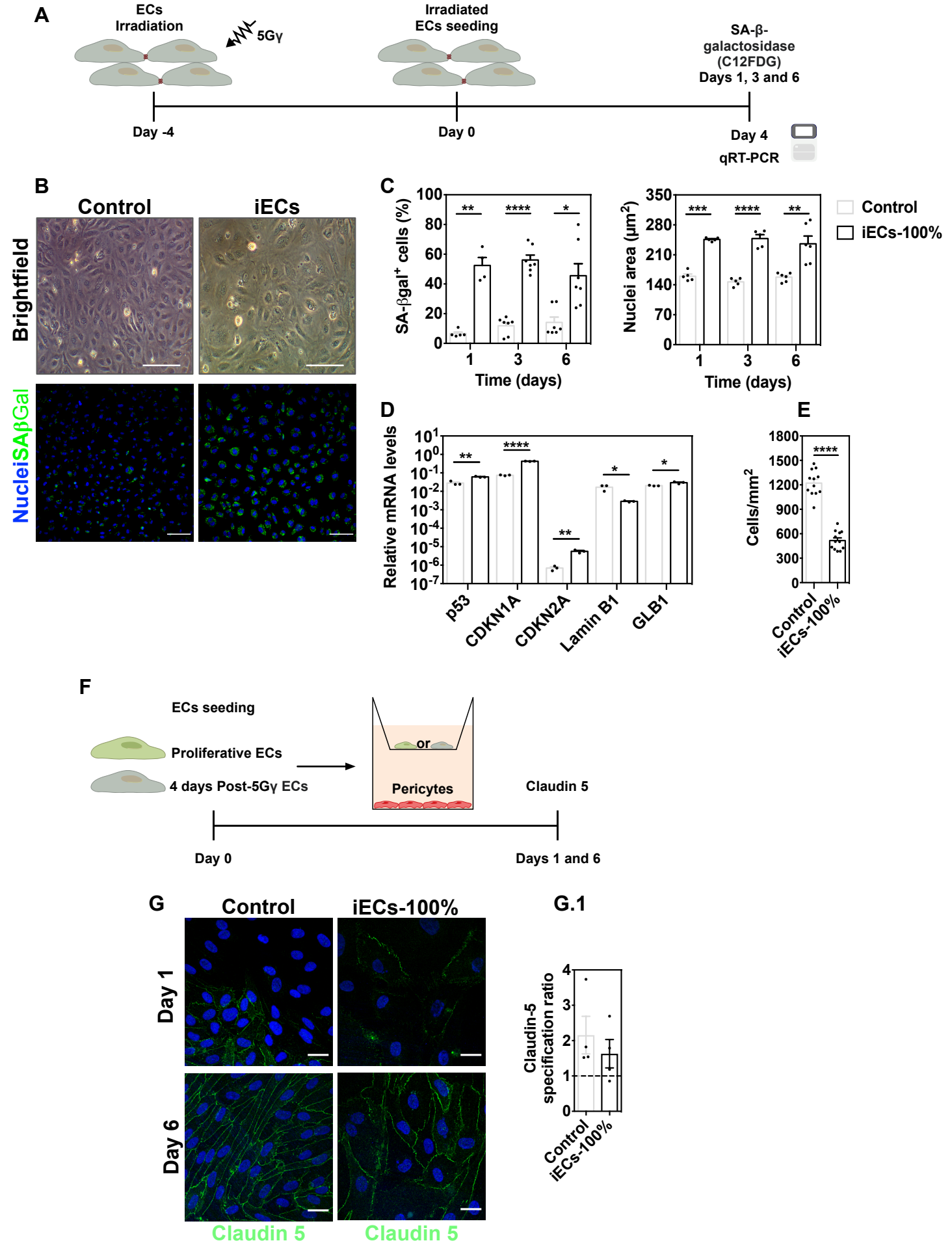

FIG S6

A

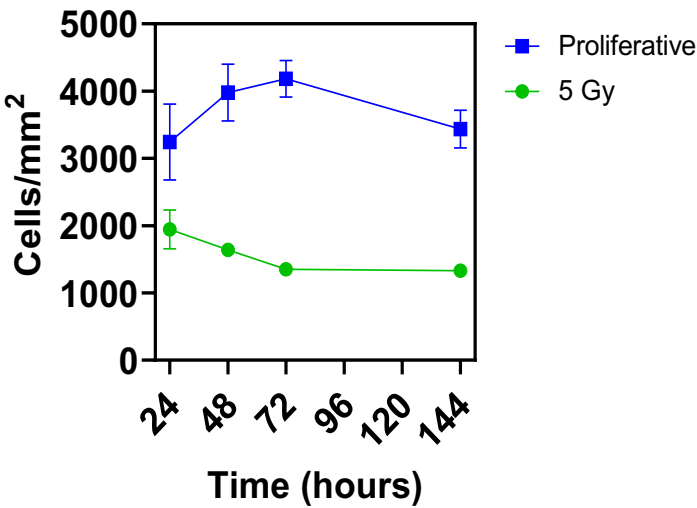

A.1

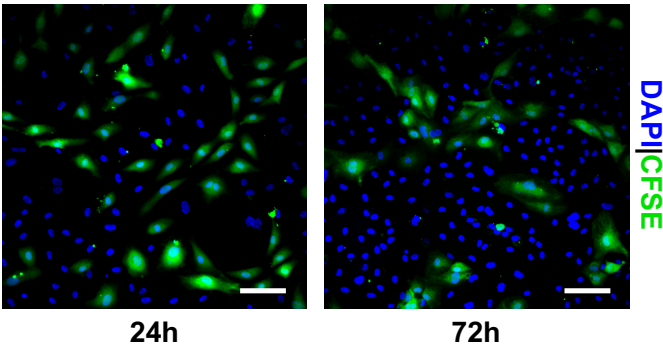

FIG S7

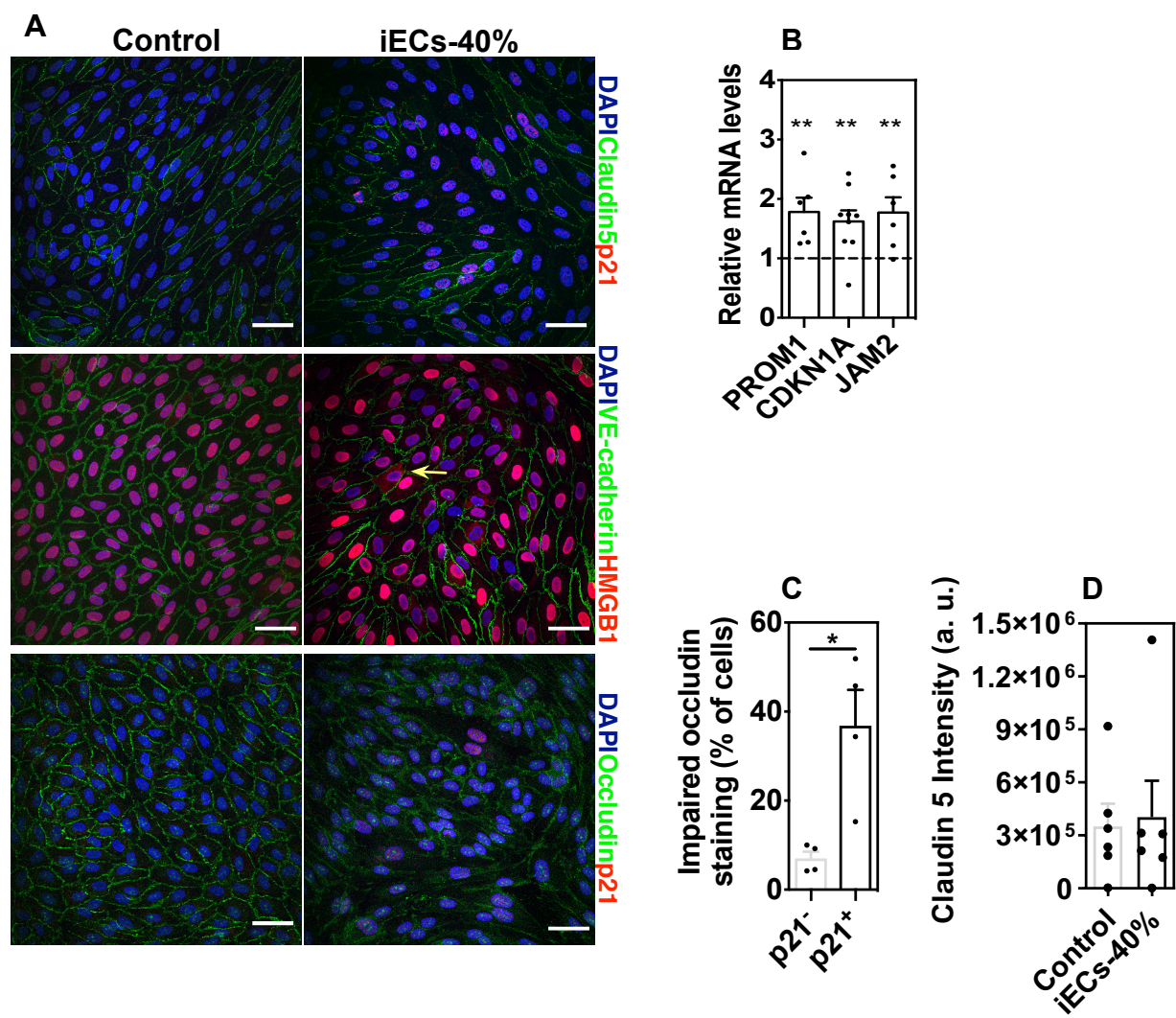

FIG S8

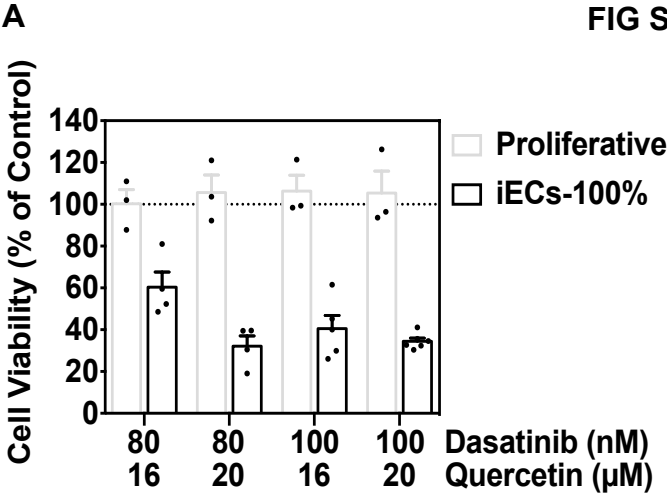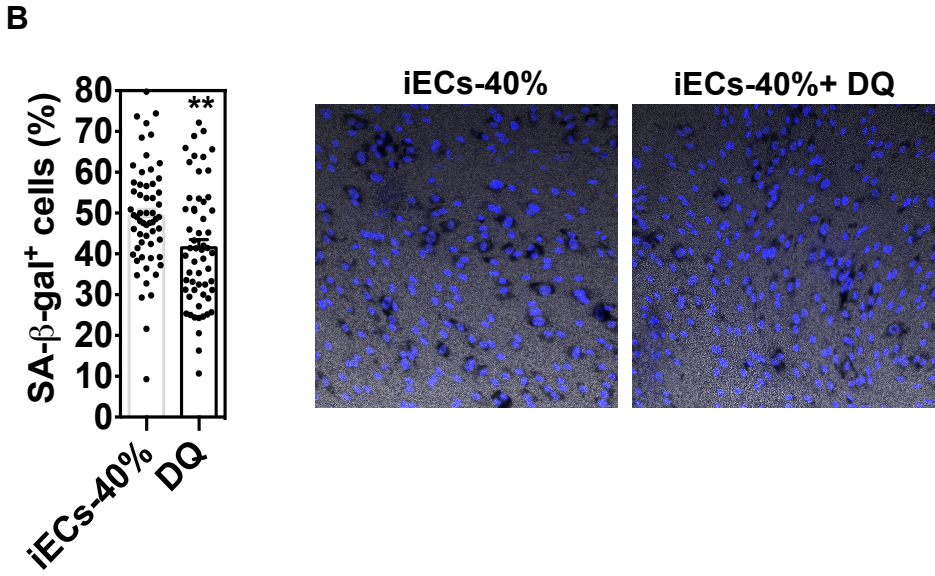

FIG S9

A

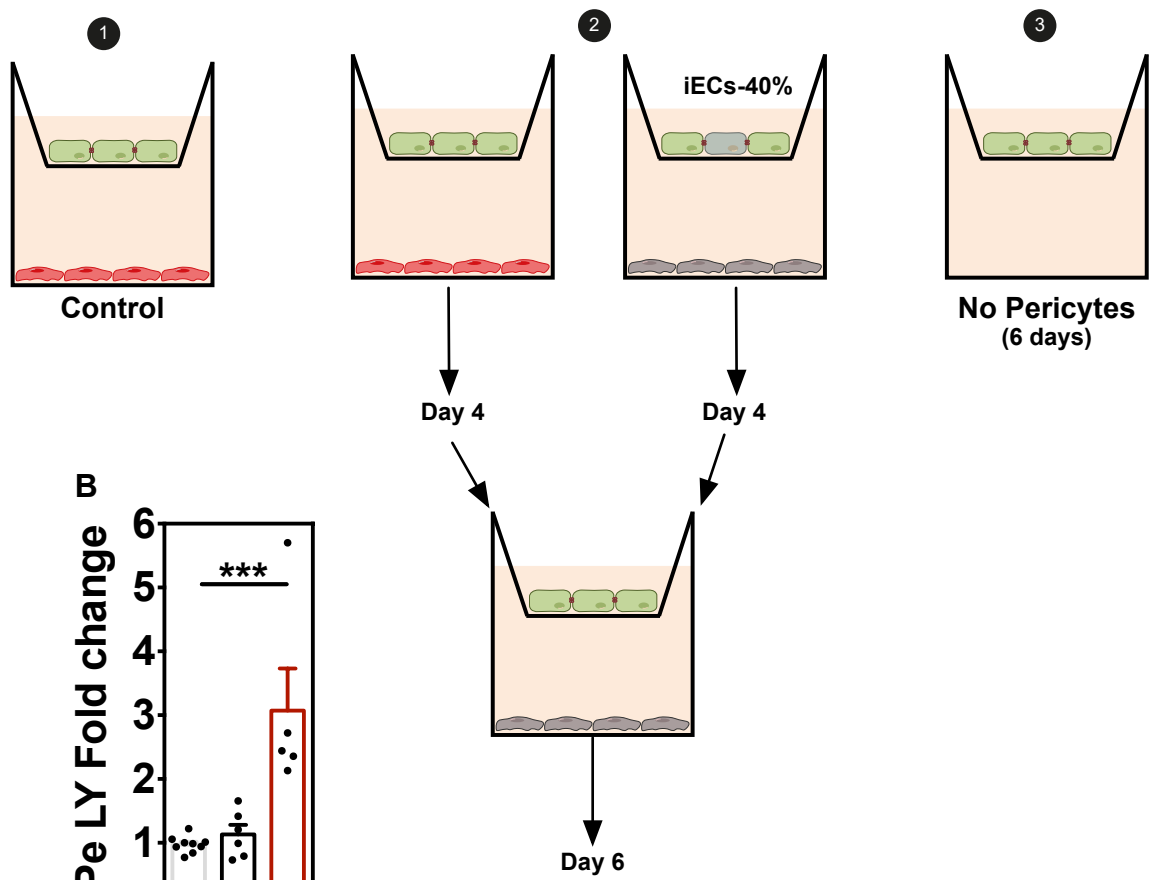

B

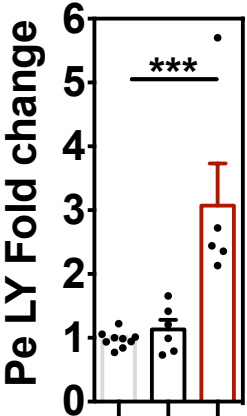

Pericytes (40%)  
Control  
No Pericytes 6d

Supplement: Supplementary file 1 — Appendix S1. [file ACEL-23-e14270-s001.pdf]
