# Supplementary material for: Blood–brain barrier dysfunction in aging is mediated by brain endothelial senescence
Source: Aging Cell. 2024 Aug 15;23(9):e14270. doi: 10.1111/acel.14270 (PMC11488312; doi:10.1111/acel.14270)
Supplement: Supplementary file 2 — Appendix S2. [file ACEL-23-e14270-s002.docx]

**Materials and Methods**

**Animals**

Experimental procedures were approved by the Institutional Animal Care and Use Committee at Mayo Clinic (protocol A26415). *INK-ATTAC^+/-^* transgenic mice were generated and genotyped as previously described ^1^. Mice were housed 2-5 *per* cage, at 22 +/- 0.5°C on a 12-12-hour day-night cycle and provided food and water *ad libitum*. Mice were allowed to age without any interventions until 4 and 24-27 months of age, when they were randomly assigned into AP20187, D+Q, or vehicle treatment groups. Mice were injected intraperitoneal (i.p.) with AP20187 or vehicle and were gavaged with Dasatinib and Quercetin in combination or with vehicle (*i.e*., each mouse received both gavage and an i.p. injection with at least one of them containing vehicle). Drugs or vehicle were administered at 9-11am for 3 consecutive days *per* week, every two weeks for the total period of 8 weeks. Concentrations of drugs and vehicle composition are listed in Table S1. We used animals from both genders: Young (3M=male; 3F=female); Aged untreated (vehicle) (8M; 6F); AP treated (8M; 2F) and DQ (7M). The protocol for the *in vivo* experiments for quantification of fluorescently labelled albumin extravasation into the brain was approved by the Portuguese DGAV 0421/000/000/2020 ORBEA 262. The animals were maintained at the animal house facility to age (22.5 months). We used animals from both genders: Young (6M;3F, 2F and 1M were used for background control); Aged (7M, 2M were used for background control)”.

**Immunohistochemistry analysis**

Cassettes with the brains were placed in 4% PFA (tissue to fixative ratio 1:3) on a shaker for no more than 24h at room temperature. Tissue was washed 2 times, 5 min with PBS under shaker. Cassettes were maintained in ethanol 70% at 4ºC that is replaced by PBS before sending to processing and paraffin embedding. The 4-μm-thick brain sections were de-paraffinized, rehydrated in descending alcohol series and washed with phosphate buffered saline (PBS). Heat mediated antigen retrieval was performed at 97ºC for 20 min using a high-temperature water bath except for albumin analysis were antigen retrieval was performed in the microwaves. If needed, sections were permeabilized with Triton X-100 (v/v Sigma-Aldrich) and then blocked for 60 min in blocking buffer at room temperature (RT) (Table S2). For Glucose transporter 1 (GLUT1) analysis, sections were incubated overnight (ON) with the primary antibody, and were then incubated with the secondary antibody Alexa Fluor® 488 goat anti-rabbit for 60 min at RT. For GFAP and GLUT1 analysis, sections were first incubated ON at 4ºC with the primary antibody against GFAP and then incubated with the secondary antibody Alexa Fluor® 488 donkey anti-goat for 60 min at RT. Subsequently sections were blocked with 5% normal goat serum (NGS) for 60 min at RT and incubated with the primary antibody for GLUT1 ON at 4ºC. On the next day, sections where incubated with the secondary antibody Cy3 Goat anti-rabbit for 60 min at RT. For HMGB1 and GLUT1 analysis, sections were first incubated ON at 4ºC with the primary antibody against HMGB1 and then incubated with the secondary antibody Cy3 AffiniPure goat anti-rabbit for 60 min at RT. For Albumin analysis sections were first incubated ON at 4ºC with the primary antibody for albumin, followed by Alexa Fluor® 488 goat anti-chicken 45 min at RT and then incubated with the primary for GLUT1 (60 min at RT) followed by the secondary antibody for GLUT1 Alexa Fluor® 555 goat anti-chicken 45 min at RT. For occludin analysis sections were incubated ON at 4ºC with the primary antibody against VE-Cadherin, on the following day with the secondary antibody Alexa Fluor® 555 donkey anti-goat for 60 min at RT. Subsequently sections were blocked in NGS for 60 min at RT and incubated ON at 4ºC with the primary antibody against occludin. On the next day sections were incubated with a biotinylated goat anti rabbit antibody (20 min RT) followed by an incubation with fluorescein avidin. After incubation with the secondary antibodies, the slices were incubated with 4′,6‐diamidine‐2′‐phenylindole dihydrochloride (DAPI, Sigma-Aldrich, St. Louis, MO, United States) for 5 min. The preparations were mounted with Vectashield HardSet Antifade Mounting Medium (Vector Laboratories, Burlingame, CA, United States).

**Immunohistochemistry image analysis**

The brain sections were visualized in the confocal microscope and from each condition, five random fields were acquired in the hippocampus area. Image J software was used for image analysis. The number of positive vessels for GLUT1 was determined. Fluorescence intensity analysis for GLUT1 and GFAP was performed using ImageJ image analysis tool set. A region of interest (ROI) was established with the threshold tool to define the specific signal for the markers analysed (discarding the associated background) and the mean fluorescence intensity was measured. For the colocalization analysis (GFAP-GLUT1), for each channel a threshold was applied to define the ROI. The ROIs of both images were combined and the area where both signals where overlaid was quantified. GLUT1^+^ cells without nuclear staining for HMGB1 were manually considered HMGB1-negative cells. For albumin analysis the threshold tool was used to define the specific signal (discarding the background assessed by the staining without the primary antibody) and the mean fluorescence intensity was measured. Fluorescence intensity analysis for occludin was performed by establishing a threshold to define the ROI corresponding to the signal for occludin (to remove the background) and the mean fluorescence intensity normalized by the area defined by the threshold was measured. To analyse the % of area of occludin coincident with vessels identified by VE-cadherin we established a threshold for each of the channels to define a ROI. The ROIs of both channels were combined and the percentage of area where both signals were overlaid was measured.

**RNAScope mRNA *in situ* hybridization assay**

Manual RNAscope assays were performed using RNAscope Multiplex Fluorescent v2 Assay Kit (Advanced Cell Diagnostics (ACD), Biotechne, Cat. No. 323100, Minneapolis, USA), according to manufacturer’s instructions. In short, 4-μm-thick brain sections were de-paraffinized, rehydrated in descending alcohol series and washed with distilled water. Target retrieval was performed for 15 min at 100°C, followed by protease treatment for 30 min at 40°C. Probes were then hybridized for 2 h at 40°C followed by RNAscope amplification. The following RNAscope probes were used: Mm Cdkn1a (p21) (Cat. No. 408551, ACD, Biotechne), p16 (Cat. No. 411011, ACD, Biotechne), and Mm occludin (Cat. No 4400401, ACD, Biotechne). The hybridization of the probe was followed by the hybridization of multiple signal amplification molecules, accordingly to the manufacturer´s protocol. The probes used were detected in the C1 probe channel which was developed using Opal 570 dye (OP-001003, Akoya Biosciences) diluted at 1:1500 in RNAscope Multiplex TSA buffer. Subsequently, sections were blocked with 5% BSA for 1 h RT and incubated with primary antibody for GLUT1 (1:250, cat. no. ab115730, Abcam). Then, they were incubated with secondary antibody Alexa Fluor® 488 Goat Anti Rabbit (1:800, Cat. No. A11034, Invitrogen). The slices were washed with PBS 1X and incubated with DAPI for 5 min. The preparations were washed with PBS 1X and mounted with Vectashield HardSet Antifade Mounting Medium (Vector Laboratories, Burlingame, CA, United States). Images were acquired with a confocal microscope (ZEISS, 710) (for p21 and ocludin sections) and an SP8 Leica (for p16 sections), and from each condition, five random fields were acquired. Image J software was used for image analysis. GLUT1^+^ cells with nuclei staining for p21, p16, and occludin probes were considered positive for these markers.

**Albumin extravasation analysis**

Albumin from bovine serum (BSA) Alexa Fluor 647 conjugate (A34785, Life Technologies) was solubilized in sterile PBS at a final concentration of 10 mg/mL. A group of young (4 months, n=5) and aged (22.5 months, n=6) C57BL6 mice were injected intravenously through the caudal vein with Alexa 647-conjugated BSA (100 μg in 100 μL of saline solution, per animal). As a control, young (n=3) and aged (n=2) mice were injected with saline solution alone to have a background measurement of the brains for the two groups of animals. The injections were performed in the absence of anaesthesia. The animals were left for 18 h, after this period the animals were sacrificed, and the brains removed. The brains were analysed *ex vivo* in IVIS optical imaging device (excitation: 640 nm; emission: Cy5.5). The average of the mean radiance and total emission measured on the saline injected mice (age associated background) was subtracted to the values measured for each one of the brains of the Alexa 647-conjugated BSA injected mice.

**Single cell RNAseq data analysis**

ScRNAseq data was analysed following the best-known practices ^2^. Data processing and visualization was performed using the Seurat package ^3^ and custom scripts in R (R: The R Project for Statistical Computing. https://www.r-project.org/). Three datasets were used herein: GSE129788 ^4^, GSE146395^5^, and GSE147693 ^6^. Datasets were filtered to exclude low-quality cells with removal of any genes detected in fewer than 3 cells and maintaining only the ones for which <5% or >95% of UMI count or gene count were attained. Datasets were subsequently normalized and scaled. Canonical Correlation Analysis (CCA)-based integration functions were used to align raw data of multiple experiments and remove batch effects. The top High-Variance Genes (HGVs) were used to find anchor genes as a stable reference to eliminate batch effect from CCA. Genes were projected into the top N component space (accessed by elbow” heuristics or the permutation-test-based jackstraw method) using Principal Component Analysis (PCA). Single cell PC scores and genes loadings were used as inputs to perform other standard dimension reduction technologies, including t-Stochastic Neighbourhood Embedding (t-SNE) and Uniform Manifold Approximation and Projection (UMAP), and search for distinct cell populations/clusters. For that, a Shared Nearest Neighbour (SNN) graph based on the Euclidean distance metric in PC space was calculated using the Louvain method. To identify cell subtypes/cell identities (neurons, astrocytes, oligodendrocytes, microglia, etc) in the GSE147693 dataset (the only one for which ECs were not clearly assigned), we employed a probabilistic method, CellAssign ^7^. Each cell was assigned to the subtype with the highest likelihood, and the algorithm will be run 3 times independently to identify and annotate clusters. Mouse Brain Atlas ^8^ or the Human Cell Atlas ^9^ was used to retrieve key genes from key cell types. After batch effect removal, quality control and cell type classification, gene count normalization and high-variance gene identification, data integration, dimensionality reduction, and clustering, we applied various methods (MAST^10^, Wilcoxon rank sum, ROC, Logistic Regression, t-test) to access Differentially Expressed Genes (DEGs) with associated adjusted p-value threshold of lower than 0.05 and requiring at least two-fold up- or downregulation in expression. All p-values were corrected for multiple testing using Benjamini-Hochberg procedure^11^. To obtain the heat-map showed in Figure S3D the list of the DEG genes resulting from the reunion of the three data bases analysed was intersected with a list of BBB enriched genes (Table S6) that was based in a previous work that analysed the expression of brain-endothelial cells versus peripheral ECs ^12^ and list of senescence related genes (Table S7) that was retrieved from “Cell Age data base”.

**Generation of the *in vitro* BBB model**

CD34^+^ cells isolation and differentiation into endothelial cells (CD34^+^-ECs) was performed as previously described with minor changes ^13^. Each CD34^+^-ECs differentiation was done from a different donor (at least 3 to 4 different donors were used for each experiment). For co-cultures, CD34^+^-ECs were used 4 days after 5 Gγ irradiation exposure (iECs) and were seeded alone or with proliferative CD34^+^-ECs (60%) at a density of 8×10^4^ cells/insert onto the Matrigel-coated (BD Biosciences) Transwell inserts (Corning Costar 3401 or 3460). iECs were seeded 4h prior to proliferative CD34^+^-ECs. Human brain vascular pericytes (HBVP) (ScienCell) were cultured in the bottom chamber according to manufacturer’s instructions. HBVP were seeded into each well of 12-well plates (Costar) at a density of 12 x 10^3^ cells/well.

**Cell Tracking**

The CellTrace™ CFSE Kit (Invitrogen, MA, USA) was used for the tracking of iECs according to manufacturer’s instructions. Briefly, iECs were incubated with 5 µM CellTrace™ CFSE in PBS 1x for 20 min at 37°C, protected from light. Then, five times the original staining volume of culture medium was added and iECs were incubated for 5 min at 37ºC. iECs were centrifuged (300 g, 5 min) and seeded. Proliferative CD34^+^-ECs were seeded 4h later without CellTrace™ CFSE. Then (24h later), cells were incubated with (0,2 µg/mL) Hoechst for 10 min at 37ºC. Cells were observed with an IN Cell Analyzer 2200 microscope (GE Healthcare, IL, USA) and, for each condition, eight fields were acquired at 24h, 48h, 72h, and 144h timepoints. IN Cell Analyzer 2200 software (GE Healthcare, IL, USA) was used to count cells and identify CFSE^+^ cells.

**Endothelial Permeability (Pe) and Transendothelial Electrical Resistance (TEER) measurements**

Pe was determined as described ^13.^ Fluorescence detection was carried out on a Synergy H1 multiplate reader (Biotek) using 432/538 nm (excitation/emission) wavelength for Lucifer yellow. Moreover, TEER was measured using a Millicell ERS-2 Epithelial Volt-Ohm Meter (Merck Millipore, Darmstadt, Germany). TEER readings of cell-free inserts were subtracted from the values obtained with cells and expressed as previously reported ^13^.

**Immunocytochemistry analysis**

After experiments, brain-like endothelial cells (day 6 of co-culture) were washed with PBS and fixed with methanol/acetone for 2 min (for Claudin-5) or PFA 4% for 10 min (for VE-Cadherin and Occludin). Cells were permeabilized for 10 min with 0.1% Triton X-100 (only for PFA 4% fixed cells) and blocked for 30 min with 5% BSA. Cells were then incubated with primary antibodies (Table S3) for 60 min at room temperature (RT) and rinsed with PBS. Next, cells were incubated with the secondary antibodies (Table S3) for 30 min at RT and washed with PBS. Afterwards, cells were permeabilized for 10 min with 1% Triton X-100 and blocked for or 30 min with 5% BSA. The cells were then incubated with primary antibodies (Table S4) ON at 4ºC and rinsed with PBS. Then, cells were incubated with the secondary antibodies (Table S4) for 30 min at RT, washed with PBS, and incubated with DAPI for 5 min. The insert filters were rinsed with PBS and mounted with Vectashield HardSet Antifade Mounting Medium (Vector Laboratories, Burlingame, CA, United States). Cells were observed with a confocal microscope, and for each condition, four random fields were acquired. Image J software was used for image analysis. For p21, the mean fluorescence intensity was determined by establishing a ROI with the threshold tool to define the specific signal for the marker (discarding the associated background) and the mean fluorescence intensity was measured. Cells above mean + 2SD fluorescence intensity were considered positive. For HMGB1, cells with HMBG1 staining in the cytoplasm were manually considered as HMGB1 extravasation cells. For Claudin-5, VE-Cadherin, and Occludin the mean fluorescence intensity was obtained.

**Colorimetric SA-β-galactosidase analysis**

The Senescence Detection Kit (Abcam, Cambrigde, UK) was used to identify SA-β-galactosidase activity of senescent cells according to manufacturer’s instructions with minor changes. Briefly, after experiments, CD34^+^ cells were washed with PBS and fixed with fixative solution for 10 min. Then, cells incubated with staining solution mix ON at 37ºC without CO_2_. After washing with PBS, cells were incubated with DAPI for 5 min. The insert filters were rinsed with PBS and mounted with Vectashield HardSet Antifade Mounting Medium. Cells were observed with and IN Cell Analyzer 2200 microscope (GE Healthcare) and, for each condition, fifteen fields were acquired. Cells with SA-β-galactosidase staining were manually considered positive.

**C 12 FDG-fluorescent SA-β-galactosidase analysis**

C_12_FDG (5-Dodecanoylaminofluorescein Di-β-D-Galactopyranoside) (Invitrogen, MA, USA) was used to identify SA-β-galactosidase activity of senescent cells according to manufacturer’s recommendations. Proliferative CD34^+^-ECs and iECs were left 1h at 37ºC without CO_2_. Then, cells were incubated with 10 µM C_12_FDG for an additional 2h at 37ºC without CO_2_. Afterwards, cells were washed with culture media and incubated with 0,2 µg/mL Hoechst for 10 minutes at 37ºC without CO_2_. After washing with PBS, cells were observed with and IN Cell Analyzer 2200 microscope (GE Healthcare) and, for each condition, fifteen fields were acquired. IN Cell Analyzer 2200 software (GE Healthcare, IL, USA) was used to count, identify and calculate the percentage of C12FDG ^+^ cells.

**Quantitative real-time PCR analysis**

After experiments, lysates were obtained using 350 μL RLT buffer (Quiagen, MD, USA) and stored at -80ºC until use. Total RNA was extracted using RNeasy Micro Kit (Qiagen) accordingly to the manufacturer instructions. Concentration and purity of extracted RNA were determined by measuring the optical density at 260, 280 and 230 nm in a NanoDrop ND-1000 spectrophotometer (NanoDrop Technologies,Inc., USA). Then, 0.3 μg of total RNA was reverse transcribed into cDNA using qScript cDNA super mix (Quanta BioSciences, MA, USA). Real-time quantitative polymerase chain reaction (qPCR) was performed using NZY Speedy qPCR Green Master Mix (nzytech) and a real-time PCR thermocycler CFX Connect Real-Time System (Bio-rad, CA, USA). qPCR reactions in triplicate were performed for each qPCR experiment. Ct data were obtained using Bio-Rad CFX Manager Software. Relative levels of mRNA were normalized to the levels of housekeeping genes *ACTB* (β-actin) or *GAPDH* mRNA according to the experiment. The qRT-PCR primer sequences can be found in Table S5.

**Statistical analysis**

All statistical tests and data analyses were performed using Prism 7 software (GraphPad Software, La Jolla, CA). Data are presented as the mean ± standard error of the mean (SEM) of sample size. The statistical tests used, and the sample size are indicated in each figure subtitle. The data obtained was checked for normality by means of the Shapiro–Wilk test and all of the data presented a normal distribution. When comparing two groups, a Student’s t test was used. The comparisons were considered statistically significant when p < 0.05.

**Figure S1- Structural and functional alterations of the mouse BBB during ageing. (A)** Brain sections (hippocampus) from young (3 months) and aged (18 months) mice: (i) representative images of the immunostaining for GLUT1 in young and aged brain sections, (ii) quantification of the number of GLUT1^+^microvessels per field and (iii) mean fluorescence intensity of the GLUT1 immunostaining. **(B)** Brain sections (hippocampus) from young (3 months) and aged (18 months) mice: (i) representative images of the immunostaining for GFAP in young and aged brain sections, (ii) quantification of the mean fluorescence intensity of GFAP staining and (iii) quantification of the area of GFAP staining co-localizing with GLUT1^+^microvessels. For each animal 3-4 different fields of the hippocampus were analyzed. Each point represents the mean of all fields analyzed per animal (5 male(M) animals for both groups, closed symbol). Results are shown as Mean ± SEM. Scale bars: 50 μm and 25 µm (crop). In A and B, statistical analyses were performed using an Unpaired Student’s t test. (**p<0.01; ***p<0.01). **(C)** Schematic representation of the experimental design. AlexaFluor 647-BSA was injected in the caudal vein of young (3 months) and aged (22.5 months) mice. Eighteen hours after the injection the animals were sacrificed, and the brains were examined ex-vivo using IVIS (exc. 640 nm; em. Cy5.5). **(C.1)** Representative images of young and old brains with and without the injection of 647-BSA. The mean radiance **(C.2)** and total emission **(C.3)** values of the brains from young and old mice not injected with 647-BSA (background) were subtracted from the data of young and old mice injected with BSA. Results shown are mean ± SEM of 5-6 animals. (Young (6M;3 female (F), 2F and 1M were used for background control); Aged (7M, 2M were used for background control, closed symbol=male and open symbol=female). Statistical analysis was performed using an Unpaired Student’s t test (*p<0.5; **p<0.01).

**Figure S2-** **BBB permeability analyses. (A, B)** Crop showing in detail a GLUT1^+^microvessel in the hippocampus of a vehicle (A) and AP (B) animal with an arbitrary line traced (yellow line). Scale bar: 20 µm. **(A.1, B.1)** Profile of GLUT-1 and albumin staining along the arbitrary line in vehicle (A.1) and AP (B.1) animal(s).

**Figure S3-** **Characterization of the senescence program in GLUT-1 negative cells (GLUT1^-^) in the hippocampus of young and aged mice.** Quantification for senescence markers p21^+^ **(A)** and p16^+^ **(B)** analyzed by RNAish in the hippocampus of young and aged mice with and without treatment. The analysis was performed in the nuclei of the cells that did not presented staining for GLUT1 and where considered non-BECs. **(A)** Quantification of the percentage of GLUT1 negative cells positive for p21 in young and aged mice with and without treatment. For each animal 3-4 different fields of the hippocampus were analyzed. Each point represents the mean of all fields analyzed per animal (4-7 different animals, all male (M), closed symbol). Results are shown as Mean ± SEM. Statistical analysis was performed using an Unpaired Student’s t test (*p<0.05; **p<0.01). **(B)** Quantification of the percentage of GLUT1 negative cells that are positive for p16 in young and aged mice with and without senolytic treatment. For each animal 3-4 different fields of the hippocampus were analyzed. Each point represents the mean of all fields analyzed per animal (young: 3 female (F) and 3M; aged with and without treatment 4-7M animals, closed symbol=male and open symbol =female). Results are shown as Mean ± SEM. Statistical analysis was performed using an Unpaired Student’s t test (*p<0.05; **p<0.01). **(C)** Quantification of the number of GLUT-1 positive nuclei per field in the hippocampus of young and aged mice with and without treatment. For each animal 3-4 different fields of the hippocampus were analyzed. Each point represents the mean of all fields analyzed per animal (young: 8 male (M), 2 female (F); aged mice with and without treatment 8-12M animals, closed symbol=male and open symbol=female). Statistical analysis was performed using an Unpaired Student’s t test (*p<0.05; ***p<0.001). **(D)** Quantification of the mean fluorescence intensity of occludin staining and quantification of the percentage of area of occludin and ve-cadherin that overlap in aged INK-ATTC brain sections with and without treatment with AP. For each animal 4-5 different fields of the hippocampus were analyzed. Each point represents the mean of all fields analyzed per animal (aged without treatment 4M and 3F, AP treated 3M and 2F, closed symbol=male and open symbol=female). Representative images of the immunostaining for occludin and ve-cadherin in aged INK-ATTC brain sections with and without treatment. Scale bar is 50 µm. **(E)** Quantification of the mean fluorescence intensity of GFAP staining and quantification of the area of GFAP staining co-localizing with GLUT1^+^microvessels. For each animal 5-6 different fields of the hippocampus were analyzed. Each point represents the mean of all fields analyzed per animal (aged without treatment 3M and 4F, AP treated 3M and 2F, closed symbol=male and open symbol=female). Representative images of the immunostaining for GFAP in aged INK-ATTC brain sections with and without treatment with AP. Scale bar is 50µm. **(D)** and **(E)** statistical analysis was performed using an Unpaired Student’s t test (not significant).

**Figure S4- Single cell RNAseq data used for the *in-silico* analysis.** **(A)** Schematic representation of the experimental design of each one of the three studies. **(B)** Table summarizing the different methodologies in the different studies. For two of the studies, unsupervised clustering has been used to identify BECs. **(C)** Venn diagrams showing the number of DEGs down- or upregulated in (i) Young *vs* Old BECs, (ii) Young *vs* Old p21^+^ BECs or (iii) Young p21^+^ vs Old p21^+^ BECs shared between the three datasets: GSE129788 (purple), GSE146395 (teal), and GSE147693 (orange). We investigated the transcriptomic profile in both p21^+^- old and p21^+^-young BECs to identify genes that were differentially expressed in both cell populations. Five mRNA transcripts (*8430408G22RIK, BTG2, CDKN1A, IER2, PLAT*) were significantly up-regulated in aged *versus* young BECs in all three datasets, of which three were transcripts well-known to be associated with cell senescence: *p21* (also known as *Cdkn1a*), tissue-type plasminogen activator and BTG anti-proliferation 2^14^. **(D)** Heatmap of the union of all senescent and BBB DEGs up- (red) and downregulated (blue) between p21^+^-young and p21^+^-old BECs, demonstrating the degree of overlap of DEGs between each dataset. Grey color refers the absence of a particular gene in the analyzed dataset. The heat-map was obtained by intersecting the reunion of all the DEGs (both up and down) from the 3 studies with a list of both BBB (162 genes, S6 SI and senescence enriched genes (279 genes, obtained from “Cell Age database”^15^, S7 SI).

**Figure S5- Preparation and characterization of an *in vitro* human senescent BBB model.** **(A)** Schematic representation of the experimental setup. CD34^+^ cells were isolated from the mononuclear fraction of human cord blood and differentiated into endothelial cells (ECs) using protocols previously described by us^13^. CD34^+^-ECs (~80% confluence) were irradiated with 5 Gγ. After changing medium cells were left for 4 days to recover. After this period, cells were either collected to extract RNA or reseeded for further characterization of the senescence program in irradiated ECs (iECs). The activity of SA-β-galactosidase was determined at days 1, 3 and 6 after replating (4, 6 and 9 after irradiation, respectively). **(B)** Representative brightfield microscopy images of CD34^+^-ECs 4 days after irradiation and fluorescence images of C12FDG assay in non-irradiated and irradiated CD34^+^-cells (iECs) at day 1, after being reseeded. Scale bar: 100 μm. **(C)** Quantification of C12FDG positive cells and analysis of nuclei area in CD34^+^-ECs and iECs. Results shown are the mean ± SEM of 4-7 independent experiments. Statistical analysis was performed using a Paired Student’s t test (*p<0.5; **p<0.01; p***<0.001 p****<0.0001). **D)** qRT-PCR analysis of non-irradiated and iECs 3 days after irradiation. Values were normalized for the reference gene *GAPDH*. Results shown are the mean ± SEM of 3 independent experiments. Statistical analysis was performed using a Paired Student’s t test (*p < 0.5; **p<0.01; p****<0.0001). **(E)** Cell density after reseeding in a transwell system with pericytes. Results shown are the mean ± SEM of 12 independent experiments. Statistical analysis was performed using a Paired Student’s t test (p****<0.0001). **(F)** Schematic representation of the experimental design. Proliferative CD34^+^-ECs or 5 Gγ irradiated CD34^+^-ECs (iECs) were seeded in a transwell system in co-culture with human pericytes for 6 days for the induction of a BEC phenotype. Because the levels of senescence at the BBB might reach 30-40% according to the *in vivo* data (Fig. 1), we co-cultured 40% iECs (at day 4 post-irradiation; according to Fig. S4C this should correspond to 18±3.3% of SA-β-Gal positive cells) plus 60% proliferative ECs (60%) with pericytes for 6 days to induce their specification. At the end of day 6, all the ECs were stained for junctional protein claudin-5. **(G)** Representative images of immunocytochemistry for junctional protein claudin-5 (green). Scale bar: 25 µm. These results indicated that the senescence program did not interfere with the induction of BEC program as demonstrated by an upregulation of claudin-5 during the co-culture period. **(G.1)** Specification ratio of iECs. The ratio values were obtained by dividing the mean fluorescence intensity (MFI) of claudin 5 at day 6 of co-culture by the MFI values at day 1. For each experiment 3-4 different fields were analyzed. Results are shown as the Mean ± SEM of 3-4 independent experiments.

**Figure S6- Tracking of proliferative ECs and iECs during culture**. iECs (at day 4 post-irradiation) were labelled with CSFE dye (green). CSFE^+^-iECs were seeded together with proliferative-ECs at a ratio of 40%-60%, respectively and maintained in culture for 6 days. Images from the same fields were acquired at 24-, 48-, 72- and 144-h timepoints. **(A)** Quantification of the number of nuclei with cytoplasm positive for CSFE dye, corresponding to iECs and number of nuclei of cells negative for CSFE dye corresponding to proliferative ECs per field. For each experiment, 8 different fields were acquired at 24-, 48-, 72- and 144-h timepoints. Results shown are Mean ± SEM of 3 independent experiments. **(B)** Representative image of the culture of 40% CSFE-iECs togheter with 60% proliferative ECs. The same field is shown after 24 and 72 h in culture. Scale bar is100 µm.

**Figure S7-** **(A)** Representative images of immunocytochemistry analysis after 6 days in culture of the BBB model with and without 40% iECs. For each experiment 3-4 different fields were analyzed. Scale bar: 50 μm**. (B)** qRT-PCR analyses of genes in BECs collected from the BBB model. The genes investigated were some of the genes that were identified in the *in-silico* analysis (see heatmap of Fig. S3D). The values presented are relative to the control condition and normalized for the housekeeping gene *ACTB* (Beta-actin). Results shown are Mean ± SEM of 6-9 independent experiments. Statistical analysis was performed using a Paired Student’s t test (**p<0.01). Our results indicate that some of the genes (PROM1, CDKN1A and JAM2) identified in the in-silico analyses were validated in our BBB model. **(C)** Quantification of the percentage of cells with impaired occludin staining within p21^-^ and p21^+^ cell populations. The occludin staining was considered impaired when irregular, blurred, or inexistent occludin staining was detected surrounding the cells. For each experiment 3-4 different fields were analyzed. Results shown are Mean ± SEM of 4 independent experiments. Statistical analysis was performed using a Paired Student’s t test (*p<0.05). **(D)** Claudin-5 mean fluorescence intensity quantification, each point represents the mean of the fields analyzed for each experiment. Results are shown as the Mean±SEM of 5 independent experiments.

**Figure S8- Effect of the senolytic combination (D+Q) in the senescent BBB *in vitro* model**. **(A)** Cellular viability was measured by Presto Blue assay in proliferative ECs and iECs. ECs were exposed to gamma irradiation (5 Gy) and left for 4 days to develop the senescence phenotype. On day 0 ECs and/or iECs were plated at a similar density and left in culture for 4 days, when combination of DQ in different concentrations were added to the culture for 24 h. After this period the medium was replaced and cell viability measured at day 6**. (B)** Quercitin 16 μM and Dasatinib 80 nM combination (affected iECs but not proliferative ECs viability) was added to the *in vitro* BBB model at day 4, for 24 h. After this period, senolytics were removed and SA-β-galactosidase activity was evaluated at day 6. Quantification of the number of SA- β -galactosidase positive cells (cells with dark staining) normalized by the total number of nuclei per field. The results are from 4 independent experiments (12-14 fields per condition were analyzed). Each dot corresponds to a different field. Statistical analysis was performed using an Unpaired Student’s t test (*p<0.05).

**Figure S9- Role of pericytes in co-culture. (A)** Schematic representation of the experiments performed. **1-**Proliferative ECs were maintained in co-culture with pericytes for 6 days (control); **2-** iECs 40% were co-cultured with pericytes for 4 days in a transwell system, in parallel, proliferative ECs were co-cultured with pericytes for 4 days. At day 4, proliferative ECs were moved to a transwell with pericytes previously exposed to iECs and co-cultured for 2 additional days; **3-** Proliferative ECs were maintained in a transwell system without pericytes. Permeability to LY was measured at day 6. **(B)** Permeability values to LY normalized to the control condition. Results are shown as the Mean ± SEM of 3 independent experiments, each experiment was performed in duplicate. Statistical analysis was performed using an Unpaired Student’s t test (***p<0.001).

Table S1- Drugs used in the mouse studies.

| **Drug** | **Dose** | **Supplier** | **Vehicle** | **Route** |
| --- | --- | --- | --- | --- |
| AP20187 | 10 mg/kg body weight | ARIAD Pharmaceuticals | PEG-400 | i.p. injection |
| Dasatinib and quercetin  (D+Q) | Dasatinib 5 mg/kg body weight  Quercetin 10 mg/kg  body weight | LC Laboratories (Dasatinib)  Sigma  (Quercetin) | 60% phosal  10% ethanol  30% PEG-400 | Oral gavage |

**Table S2- List of antibodies for immunohistochemistry analysis.**

| **Antibody** | **Dilution** | **Host** | **Supplier** | **Catalog number** | **Buffer for Antigen Retrieval** | **Permeabilization** | **Blocking** |
| --- | --- | --- | --- | --- | --- | --- | --- |
| **Primary antibodies** | | | | | | | |
| HMGB1 | 1:250 | rabbit | Abcam | ab18256 | Citrate pH=6 | 10 min 1% Triton X-100 RT | 5% BSA |
| GLUT1 | 1:250 | rabbit | Abcam | ab115730 | TRIS EDTA pH=9 or  Citrate pH=6 | 10 min 0.1% Triton X-100 RT | 5% NGS or  1:60 NGS in 0.1 % BSA/PBS |
| GFAP | 4 µm/mL | goat | Abcam | ab53554 | Citrate pH=6 | 15 min 0.5% Triton X-100 RT | 5% NDS |
| Albumin | 1:100 | chicken | Abcam | Ab106582 | Citrate pH=6 | 10 min 0.1% Triton X-100 RT | 1:60 NGS in 0.1 % BSA/PBS |
| Occludin | 1:100 | rabbit | Abcam | AB216327 | TRIS EDTA pH=9 | 10 min 0.1% Triton X-100 RT | 1:60 NGS in 0.1 % BSA/PBS |
| VE-Cadherin | 1:100 | goat | RD systems | AF1002 | TRIS EDTA pH=9 | - | antibody dilutent with background reducing agent  DAKO  S3022 |
| **Secondary antibodies** | | | | | | | |
| anti-goat Alexa Fluor 488 | 1:800 | donkey | Invitrogen | A11055 | - | - | 1% BSA |
| anti-rabbit Alexa Fluor 488 | 1:800 | goat | Invitrogen | A11034 | - | - | 5% BSA |
| anti-mouse Alexa Fluor 488 | 1:800 | donkey | Invitrogen | A21202 | - | - | 1% BSA |
| anti-mouse Alexa Fluor 555 | 1:800 | goat | Invitrogen | A21422 | - | - | 1% BSA |
| anti-rabbit Cy3 | 1:800 | goat | Jackson Immuno  Research | 111-165-144 | - | - | 5% BSA |
| Anti-chicken Alexa 488 | 1:1000 | goat | Abcam | A11042 | - | - | 1:60 NGS in 0.1 % BSA/PBS |
| anti-goat  Alexa Fluor-555 |  | donkey | Invitrogen |  | - | - | antibody dilutent with background reducing agent  DAKO  S3022 |
| anti-rabbit IgG (H+L), biotinylated | 1:200 | goat | Vector Laboratories | BA-100-1.5 | - | - | 1:60 NGS in 0.1 % BSA/PBS |
| Fluorescein-avidin DCS | 1:500 | - | Vector Laboratories | A-2011-1 | - | - | PBS |

NDS, normal donkey serum; NGS, normal goat serum

**Table S3- List of antibodies for immunocytochemistry analysis for junctional proteins.**

| **Antibody** | **Dilution** | **Host** | **Supplier** | **Catalog number** |
| --- | --- | --- | --- | --- |
| **Primary antibodies** | | | | |
| Claudin-5 | 1:100 | rabbit | Thermo Fisher Scientific | 341600 |
| VE-Cadherin | 1:50 | mouse | Santa Cruz Biotechnology | sc-9989 |
| Occludin | 1:125 | rabbit | Thermo Fisher Scientific | 711500 |
| **Secondary antibodies** | | | | |
| anti-rabbit Alexa Fluor 488 | 1:800 | goat | Invitrogen | A11034 |
| anti-mouse Alexa Fluor 488 | 1:800 | donkey | Invitrogen | A21202 |

**Table S4- List of antibodies for immunocytochemistry analysis of senescence markers.**

| **Antibody** | **Dilution** | **Host** | **Supplier** | **Catalog number** |
| --- | --- | --- | --- | --- |
| **Primary antibodies** | | | | |
| p21 | 1:50 | mouse | Santa Cruz Biotechnology | sc-6246 |
| HMGB1 | 1:500 | rabbit | Abcam | ab18256 |
| **Secondary antibodies** | | | | |
| anti-mouse Alexa Fluor 555 | 1:800 | goat | Invitrogen | A21422 |
| anti-rabbit Cy3 | 1:800 | goat | Jackson Immuno  Research | 111-165-144 |

**Table S5- List of primers and sequences used in qRT-PCR analysis.**

| **Gene** | **Primer** | **Sequence (5'-3')** |
| --- | --- | --- |
| *RAD21* | Forward | GTGGAAAGAGACAGGAGGAGTAG |
| *RAD21* | Reverse | AGGTCTTCTGGTACAAGCGGTG |
| *JAM2* | Forward | AGACTTGGCTCCCAAAGCACCA |
| *JAM2* | Reverse | TTCCCAGGACACCTGCGATATC |
| *PROM1* | Forward | ACAACACTACCAAGGACAA |
| *PROM1* | Reverse | GGACTTAATCTCATCAAGAACAG |
| *CDKN1A* | Forward | CAGCATGACAGATTTCTACC |
| *CDKN1A* | Reverse | CAGGGTATGTACATGAGGAG |
| *OCLN* | Forward | TTCTGGATCTCTATATGGTTCA |
| *OCLN* | Reverse | CCACAACACAGTAGTGATAC |
| *SOD1* | Forward | GAGCAGAAGGAAAGTAATGG |
| *SOD1* | Reverse | GATTAAAGTGAGGACCTGC |
| *CDKN2A* | Forward | AGCATGGAGCCTTCG |
| *CDKN2A* | Reverse | ATCATGACCTGGATCGG |
| *LMNB1* | Forward | AAGGCGAAGAAGAGAGGTTGAAG |
| *LMNB1* | Reverse | GCGGAATGAGAGATGCTAACACT |
| *p53* | Forward | GAAGAGAATCTCCGCAAGA |
| *p53* | Reverse | CGGATCTGAAGGGTGAAA |
| *GLB1* | Forward | GACAGTACCAGTTTTCTGAG |
| *GLB1* | Reverse | ATAGACTCTTTCTCTAGCAGC |
| *ACTB* | Forward | ACTCTTCCAGCCTTCCTTC |
| *ACTB* | Reverse | GATGTCCACGTCACACTTC |

**Table S6- List of BBB-enriched genes used in the *in silico* analysis** ^12^.

| Apc | Cldn5 | Magi3 | Slc38a3 |
| --- | --- | --- | --- |
| 9430020k01rik | Ctnnb1 | Mfsd2a | Slc39a10 |
| A930038c07rik | Cxcl12 | Ocln | Slc6a6 |
| Abcb1a | Cyth3 | Pdcd6ip | Slc7a1 |
| Abcc4 | Efr3b | Pecam1 | Slc7a5 |
| Abcg2 | Esam | Pglyrp1 | Slco1a2 |
| Abhd2 | F11r | Pltp | Slco1a4 |
| Ablim1 | Flt1 | Pltp | Slco1c1 |
| Amot | Fosb | Pmp22 | Sparcl1 |
| Amotl1 | Foxf2 | Prom1 | Spock2 |
| Amotl2 | Foxq1 | Ptn | Stra6 |
| Apcdd1 | Frmd4b | Rapgef2 | Tfrc |
| Apod | Gpcpd1 | Sgpp2 | Tjp1 |
| Arhgef2 | Hiat1 | Sipa1l3 | Tjp2 |
| Ash1l | Igf1r | Slc16a2 | Tnfrsf19 |
| Atp10a | Itih5 | Slc16a4 | Ubn1 |
| Bsg | Itm2a | Slc19a3 | Usp53 |
| Cav1 | Jam2 | Slc1a1 | Vwa1 |
| Ccdc141 | Lef1 | Slc22a8 | Vwf |
| Cdh5 | Lrp8 | Slc2a1 | Zic2 |
| Cgnl1 | Lsr | Slc301a | Zic3 |

**Table S7- List of ageing genes used in the *in silico* analysis retrieved from Cell Age database.**

| AAK1 | FASTK | MCRS1 | SENP2 |
| --- | --- | --- | --- |
| ABI3 | FBXO31 | MDH1 | SENP7 |
| ACLY | FOS | MECP2 | SERPINE1 |
| ADCK5 | FOXM1 | MMP9 | SFN |
| AGT | FOXO3 | MOB3A | SGK1 |
| AKR1B1 | FXR1 | MORC3 | SIK1 |
| AKT1 | G6PD | MORF4 | SIN3B |
| ALOX15B | GAPDH | MVK | SIRT1 |
| AR | GATA4 | MXD4 | SIRT6 |
| ARPC1B | GKN1 | MYC | SIX1 |
| ASF1A | GLB1 | MYLK | SLC13A3 |
| ASPH | GNG11 | NADK | SLC16A7 |
| ATF7IP | GRK6 | NANOG | SMARCA4 |
| ATM | HDAC1 | NDRG1 | SMARCB1 |
| AURKA | HDAC4 | NEK1 | SMG1 |
| AXL | HEPACAM | NEK4 | SMURF2 |
| BAG3 | HIVEP1 | NEK6 | SNAI1 |
| BCL6 | HJURP | NFE2L2 | SOCS1 |
| BHLHE40 | HK3 | NINJ1 | SOD1 |
| BLK | HMGB1 | NOTCH3 | SORBS2 |
| BLVRA | HRAS | NOX4 | SOX2 |
| BMI1 | HSPA5 | NR2E1 | SOX5 |
| BRAF | HSPB2 | NTN4 | SP1 |
| BRCA1 | ID1 | NUAK1 | SPIN1 |
| BRD7 | ID4 | OTX2 | SPOP |
| BTG3 | IFNG | P3H1 | SRC |
| C11orf31 | IGFBP1 | PAK4 | SREBF1 |
| CAV1 | IGFBP3 | PATZ1 | SRSF1 |
| CBX7 | IGFBP5 | PBRM1 | STAT5B |
| CBX8 | IGFBP6 | PCGF2 | STK32C |
| CCND1 | IL1A | PDCD10 | STK40 |
| CDK1 | IL8 | PDIK1L | SUPT5H |
| CDK18 | ING1 | PDPK1 | SYK |
| CDK2AP1 | ING2 | PDZD2 | TACC3 |
| CDK4 | IRF3 | PEBP1 | TBX2 |
| CDK6 | IRF5 | PEX19 | TERC |
| CDKN1A | IRF7 | PIAS4 | TERF2 |
| CDKN1B | ITGB4 | PIK3C2A | TERT |
| CDKN1C | ITPK1 | PIK3R5 | TFAP4 |
| CDKN2A | ITPKB | PIM1 | TFDP1 |
| CDKN2AIP | ITSN2 | PKM | TGFB1I1 |
| CDKN2B | KCNJ12 | PLA2R1 | TLR3 |
| CEBPB | KDM4A | PML | TMSB4X |
| CENPA | KDM5B | PMVK | TNFSF13 |
| CHEK1 | KIAA1524 | PNPT1 | TNFSF15 |
| CKB | KL | POT1 | TOP1 |
| CPEB1 | KSR2 | POU5F1 | TP53 |
| CSNK1A1 | LATS1 | PPM1B | TP63 |
| CSNK2A1 | LEO1 | PPM1D | TPR |
| CTNNAL1 | LGALS3 | PRKCD | TRIM28 |
| CXCL1 | LIMA1 | PRKCH | TRPM8 |
| CYR61 | LIMK1 | PRMT6 | TXN |
| DDB2 | MAD2L1 | PROX1 | TXNIP |
| DEK | MAGEA2 | PRPF19 | TYK2 |
| DGCR8 | MAGOH | PSMB5 | UBTD1 |
| DHCR24 | MAGOHB | PSMD14 | USP1 |
| DHX9 | MAP2K1 | PTRF | VEGFA |
| DLX2 | MAP2K2 | PTTG1 | VENTX |
| DPY30 | MAP2K3 | RAD21 | WNT16 |
| DUSP16 | MAP2K6 | RAF1 | WNT2 |
| DUSP3 | MAP2K7 | RB1 | WRN |
| E2F1 | MAP3K6 | RBP2 | WT1 |
| EHF | MAP3K7 | RBX1 | WWP1 |
| ENDOG | MAP4K1 | RNASEL | XAF1 |
| EPHA3 | MAPK12 | RPS6KA6 | YAP1 |
| ERRFI1 | MAPK14 | RSL1D1 | YPEL3 |
| ETS1 | MAPKAPK5 | RUNX1 | ZFP36 |
| ETS2 | MAST1 | RUVBL2 | ZMAT3 |
| EWSR1 | MATK | SENP1 | ZNF148 |
| EZH2 | MCL1 |  |  |

**References:**

1 Baker, D. J. *et al.* Clearance of p16Ink4a-positive senescent cells delays ageing-associated disorders. *Nature* **479**, 232-236 (2011). <https://doi.org:10.1038/nature10600>

2 Luecken, M. D. & Theis, F. J. Current best practices in single-cell RNA-seq analysis: a tutorial. *Molecular systems biology* **15**, e8746 (2019). <https://doi.org:10.15252/msb.20188746>

3 Butler, A., Hoffman, P., Smibert, P., Papalexi, E. & Satija, R. Integrating single-cell transcriptomic data across different conditions, technologies, and species. *Nature Biotechnology* **36**, 411-420 (2018). <https://doi.org:10.1038/nbt.4096>

4 Ximerakis, M. *et al.* Single-cell transcriptomic profiling of the aging mouse brain. *Nat Neurosci* **22**, 1696-1708 (2019). <https://doi.org:10.1038/s41593-019-0491-3>

5 Chen, M. B. *et al.* Brain Endothelial Cells Are Exquisite Sensors of Age-Related Circulatory Cues. *Cell Rep* **30**, 4418-4432 e4414 (2020). <https://doi.org:10.1016/j.celrep.2020.03.012>

6 Zhao, L. *et al.* Pharmacologically reversible zonation-dependent endothelial cell transcriptomic changes with neurodegenerative disease associations in the aged brain. *Nat Commun* **11**, 4413 (2020). <https://doi.org:10.1038/s41467-020-18249-3>

7 Zhang, A. W. *et al.* Probabilistic cell-type assignment of single-cell RNA-seq for tumor microenvironment profiling. *Nature methods* **16**, 1007-1015 (2019). <https://doi.org:10.1038/s41592-019-0529-1>

8 Islam, S. *et al.* Quantitative single-cell RNA-seq with unique molecular identifiers. *Nature methods* **11**, 163-166 (2014). <https://doi.org:10.1038/nmeth.2772>

9 Regev, A. *et al.* The Human Cell Atlas. *eLife* **6** (2017). <https://doi.org:10.7554/eLife.27041>

10 Finak, G. *et al.* MAST: a flexible statistical framework for assessing transcriptional changes and characterizing heterogeneity in single-cell RNA sequencing data. *Genome Biology* **16**, 278 (2015). <https://doi.org:10.1186/s13059-015-0844-5>

11 Haynes, W. in *Encyclopedia of Systems Biology* (eds Werner Dubitzky, Olaf Wolkenhauer, Kwang-Hyun Cho, & Hiroki Yokota) 78-78 (Springer New York, 2013).

12 Munji, R. N. *et al.* Profiling the mouse brain endothelial transcriptome in health and disease models reveals a core blood-brain barrier dysfunction module. *Nat Neurosci* **22**, 1892-1902 (2019). <https://doi.org:10.1038/s41593-019-0497-x>

13 Cecchelli, R. *et al.* A stable and reproducible human blood-brain barrier model derived from hematopoietic stem cells. *PLoS One* **9**, e99733 (2014). <https://doi.org:10.1371/journal.pone.0099733>

14 West, M. D., Shay, J. W., Wright, W. E. & Linskens, M. H. Altered expression of plasminogen activator and plasminogen activator inhibitor during cellular senescence. *Exp Gerontol* **31**, 175-193 (1996). <https://doi.org:10.1016/0531-5565(95)02013-6>

15 *CellAge: The Database of Cell Senescence Genes*, <<https://genomics.senescence.info/cells/>> (2022).
